# ARL5B Drives Esophageal Squamous Cell Carcinoma Progression via ROCK1–SREBP1‐Mediated Lipid Metabolic Reprogramming

**DOI:** 10.1002/advs.202512895

**Published:** 2025-10-27

**Authors:** Xinyue Ma, Yanfei Sun, Hongyuan Mao, Chenhan Huang, Zerun Li, Tianzi Wang, Dizhi Jiang, Xinyu Zhang, Zhenyu Yuan, Zhihui Zhang, Bo Cheng, Ruiqing Wang, Yufeng Cheng

**Affiliations:** ^1^ Qilu Hospital of Shandong University Cheeloo College of Medicine Shandong University Jinan 250012 China; ^2^ Department of Neurosurgery, Qilu Hospital, Cheeloo College of Medicine and Institute of Brain and Brain‐Inspired Science Shandong University Jinan 250012 China; ^3^ Jinan Microecological Biomedicine Shandong Laboratory and Shandong Key Laboratory of Brain Health and Function Remodeling Jinan 250117 China; ^4^ Institute of Marine Science and Technology Shandong University Qingdao 266237 China; ^5^ Radiology Department, National Cancer Center Chinese Academy of Medical Sciences and Peking Union Medical College Beijing 100021 China

**Keywords:** ARL5B, ESCC, lipid metabolism, ROCK1, SREBP1

## Abstract

Esophageal squamous cell carcinoma (ESCC) remains a highly aggressive malignancy with a 5 year survival rate below 30%, underscoring the urgent need for targeted therapeutic approaches. Here adenosine diphosphate (ADP)‐ribosylation factor‐like protein 5B (ARL5B) is identified as a key candidate oncogene that drives ESCC progression by modulating lipid metabolism via the ras homologous‐associated coiled‐coil containing protein kinase 1(ROCK1)–sterol regulatory element‐binding protein 1 (SREBP1) signaling axis. Through the Cancer Genome Atlas (TCGA) pan‐cancer analysis, ARL5B is initially identified as a promising candidate gene, correlating with advanced tumor, node, metastasis (TNM) stages and poor survival. Functional assays demonstrate that ARL5B knockdown significantly suppresses cell proliferation, invasion, and growth in vivo, while promoting apoptosis. Mechanistically, ARL5B facilitates the activation and nuclear translocation of SREBP1 through ROCK1, thereby enhancing lipogenic programming. Finally, pharmacological inhibition of either ROCK1 or SREBP1 abrogates the oncogenic effects induced by ARL5B overexpression, confirming the functional dependency on this pathway. These results establish ARL5B as a central regulator of lipid metabolism in ESCC and highlight its potential as a therapeutic target for precision oncology.

## Graphical abstract

Esophageal squamous cell carcinoma urgently requires new therapeutic strategies. Adenosine diphosphate‐ribosylation factor‐like protein 5B (ARL5B) is identified as a novel oncogene that remodels fatty acid metabolism through the ras homologous‐associated coiled‐coil containing protein kinase 1 (ROCK1)–sterol regulatory element‐binding protein 1 (SREBP1) axis, driving proliferation and lipid reprogramming, and representing a promising target for precision oncology.

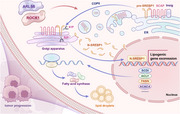

## 1 Introduction

Esophageal squamous cell carcinoma (ESCC), the most prevalent histological subtype of esophageal cancer, constitutes ≈90% of global esophageal malignancy cases.^[^ ^1^, ^2^ ^]^ This aggressive tumor is notorious for its rapid progression and limited therapeutic options, resulting in a dismal prognosis with a 5 year survival rate of merely 15–25%.^[^ ^3^, ^4^ ^]^ While molecularly targeted therapies have shown partial success, the lack of effective precision treatment targets continues to hinder clinical outcomes for the majority of patients.^[^ ^5^, ^6^ ^]^ Consequently, there is an urgent need to identify key mediators of ESCC progression and elucidate their regulatory mechanisms.

The adenosine diphosphate (ADP) ‐ ribosylation factor (ARF) ‐ like small GTPase (ARL) family has recently garnered attention in cancer biology.^[^ ^7^, ^8^, ^9^, ^10^, ^11^ ^]^ As a key member of this family, ADP‐ribosylation factor‐like protein 5B (ARL5B) plays critical roles in vesicular trafficking, organelle distribution, and metabolic regulation.^[^ ^12^, ^13^ ^]^ Emerging evidence indicates that ARL5B promotes tumor cell invasion and migration in breast and prostate cancers through modulating lysosomal positioning or inducing secretion of metabolic factors.^[^ ^14^, ^15^, ^16^, ^17^ ^]^ However, systematic investigations into the expression profile, functional role, and underlying mechanisms of ARL5B in ESCC remain lacking, including whether it regulates metabolic states of ESCC, remains unexplored territory. This study pioneers the systematic elucidation of ARL5B's overexpression in ESCC through clinical specimen and cellular model analyses, and evaluates its functional contributions to tumor progression to address this scientific void.

Intriguingly, recent studies have begun to illuminate ARL5B's underappreciated role in metabolic regulation. Given its established role in intracellular cargo transport^[^ ^12^, ^13^ ^]^—a process intricately linked to cellular metabolic activity—and may particularly participate in the spatial regulation of energy metabolism and lipid synthesis. Although existing literature suggests ARL5B‐driven metabolic alterations in other malignancies,^[^ ^15^, ^18^, ^19^ ^]^ its direct involvement in lipid metabolism, a core adaptive mechanism in cancer, remains uninvestigated. Exploring whether ARL5B contributes to ESCC progression via lipid metabolic reprogramming thus carries significant scientific implications.

Metabolic reprogramming represents a cornerstone of cancer cell survival, enabling adaptation to nutrient‐scarce microenvironments and relentless proliferation demands. In ESCC, lipid metabolic alterations are prominent, fulfilling dual roles as both structural precursors for membrane biosynthesis and bioactive mediators of signaling cascades, energy homeostasis, and immune evasion.^[^ ^20^, ^21^, ^22^ ^]^ De novo lipogenesis, as the initiating step and central regulatory node within the lipid metabolic network, is typically subjected to precise regulation by transcription factors including sterol regulatory element‐binding protein 1 (SREBP1).^[^ ^23^ ^]^ Nevertheless, the upstream regulatory signals activating lipogenesis in ESCC remain poorly characterized, with the interplay between small GTPases and these pathways yet to be elucidated.

Our integrated approach combining multiomics data analysis and functional experiments demonstrated that ARL5B is not only markedly upregulated in ESCC tissues but also correlates significantly with poor clinical prognosis in patients. Functionally, ARL5B enhances ESCC cell proliferation, migratory capacity, and intracellular lipid accumulation. Mechanistically, we identified that ARL5B interacts directly with ROCK1, thereby facilitating both Golgi apparatus trafficking and proteolytic activation of SREBP1. This activation drives the transcriptional upregulation of key lipogenic enzymes, including fatty acid synthase (FASN) and stearoyl‐CoA desaturase 1 (SCD1), thereby amplifying de novo lipid biosynthesis. Importantly, this study delineates a novel regulatory axis through which ARL5B orchestrates metabolic reprogramming in ESCC, bridging oncogenic signaling with lipid metabolic dysregulation. Beyond elucidating ARL5B's pivotal role in tumor lipid metabolism, our findings establish a theoretical framework for targeting this pathway and propose ARL5B as a potential therapeutic vulnerability for metabolic intervention strategies in ESCC.

## 2 Results

### 2.1 ARL5B Expression Is Upregulated in ESCC and Correlates with Disease Progression

**Figure 1:**
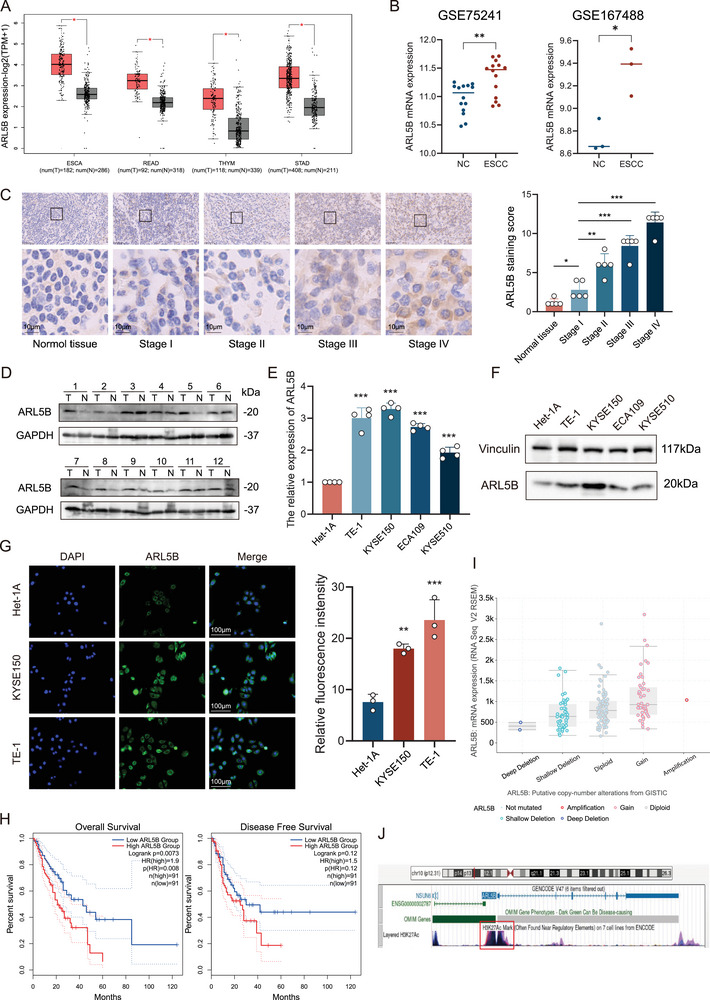
ARL5B expression is upregulated in ESCC and correlates with disease progression. A) Relative mRNA levels of ARL5B in tumors including esophageal carcinoma, rectum adenocarcinoma (READ), thymoma (THYM), and stomach adenocarcinoma (STAD) based on the transcriptome sequencing data of TCGA database. B) Relative mRNA expression of ARL5B in ESCC tissues versus adjacent nontumorous tissues from GEO database (accession numbers: GSE167488 and GSE75241). C) Representative immunohistochemical (IHC) images of ARL5B staining intensity and corresponding quantitative analysis (*H*‐scores) in normal esophageal tissues and tumor tissues grouped into stages I to IV (*n* = 5 per group). Scale bar: 50 µm (top) and 10 µm (bottom). D) Western blot analysis of ARL5B protein levels in 12 pairs of ESCC tumor tissues (T) and matched adjacent normal tissues (N). E,F) qRT‐PCR analysis of E) ARL5B mRNA levels and F) western blot analysis of ARL5B protein in the normal esophageal epithelial cell line Het‐1A compared to four ESCC cell lines (TE‐1, KYSE150, ECA109, and KYSE510). G) Representative immunofluorescence (IF) images showing cytoplasmic ARL5B staining in Het‐1A, KYSE150, and TE‐1 cells. Right, quantification of fluorescence intensity with ImageJ. Scale bar: 100 µm. H) Kaplan–Meier plot depicting overall survival and disease‐free survival in a cohort of 182 ESCA patients stratified by high versus low ARL5B expression. I,J) Correlation plots of ARL5B mRNA expression levels against I) copy number alterations and J) H3K27ac promoter modification signals, representing genetic and epigenetic associations. Data are presented as mean SD. *, *p* < 0.05; **, *p* < 0.01; ***, *p* < 0.001; and ns, no significance.

Bioinformatic analysis based on the Cancer Genome Atlas (TCGA) database initially revealed a significant association between ARL5B expression and multiple malignancies, including esophageal carcinoma (ESCA), rectum adenocarcinoma (READ), thymoma (THYM), stomach adenocarcinoma (STAD). Comparative analysis of 182 ESCA specimens and 286 adjacent normal esophageal epithelial tissues demonstrated a marked upregulation of ARL5B messenger RNA (mRNA) levels in tumor tissues (*p* < 0.05; **Figure** **1**A; Figure S1A, Supporting Information), implicating ARL5B as a potential oncogenic driver in ESCA. To further identify the clinical significance of ARL5B expression, we investigated ESCC cohorts from the Gene Expression Omnibus (GEO) database. ARL5B expression was consistently and significantly elevated in ESCC compared to adjacent nontumorous tissues (Figure 1B). To corroborate these findings, we initiated immunohistochemistry (IHC) analysis using normal tissues and ESCC specimens from histological stages I–IV (*n* = 5 per group). Quantitative analysis revealed consistently higher ARL5B expression in ESCC tissues compared to adjacent normal epithelia (Figure 1C, left). ARL5B expression exhibited a stepwise elevation across tumor stages (H‐score analysis, normal tissue vs stage I, *p* < 0.05; stage I vs stage II, *p* < 0.01; stage I vs stage III, *p* < 0.001; stage I vs stage IV, *p* < 0.001), with stage IV lesions showing the most intense staining (Figure 1C, right). Immunoblotting assays of 12 paired ESCC tumor and normal tissue samples demonstrated significantly elevated ARL5B protein levels in tumor tissues relative to normal counterparts (Figure 1D). In cellular models, parallel analyses using quantitative reverse transcription polymerase chain reaction  (qRT‐PCR) and western blotting in normal esophageal epithelial cell line Het‐1A versus four ESCC cell lines (TE‐1, KYSE150, ECA109, and KYSE510) demonstrated concordant upregulation of ARL5B at both transcriptional (*p* < 0.01) and translational levels (*p* < 0.01) in malignant cells (Figure 1E,F). Subsequent immunofluorescence (IF) staining revealed intensified cytoplasmic ARL5B signals in KYSE150 and TE‐1 cells compared to Het‐1A (*p* < 0.01 and *p* < 0.001 respectively; Figure 1G), highlighting ARL5B overexpression in ESCC. Clinically, Kaplan–Meier survival analysis of 182 ESCA patients with available followup data uncovered a striking prognostic impact: patients with high ARL5B expression exhibited significantly shorter overall survival compared to low expressers (Hazard ratio (HR) = 1.9, *p* = 0.008; Figure 1H). ARL5B expression was positively correlated with copy number alterations, which also promoted by H3K27ac modification. This suggests that the high expression of ARL5B in ESCC is influenced by genetic and epigenetic factors (Figure 1I,J).

Collectively, these multiplatform investigations integrating bioinformatic prediction with histological validation, cellular characterization, and clinical correlation systematically establish ARL5B as a pathologically upregulated molecule in ESCC, providing critical foundation for subsequent mechanistic inquiries.

### 2.2 ARL5B Promotes ESCC Proliferation and Migration In Vitro and In Vivo

**Figure 2:**
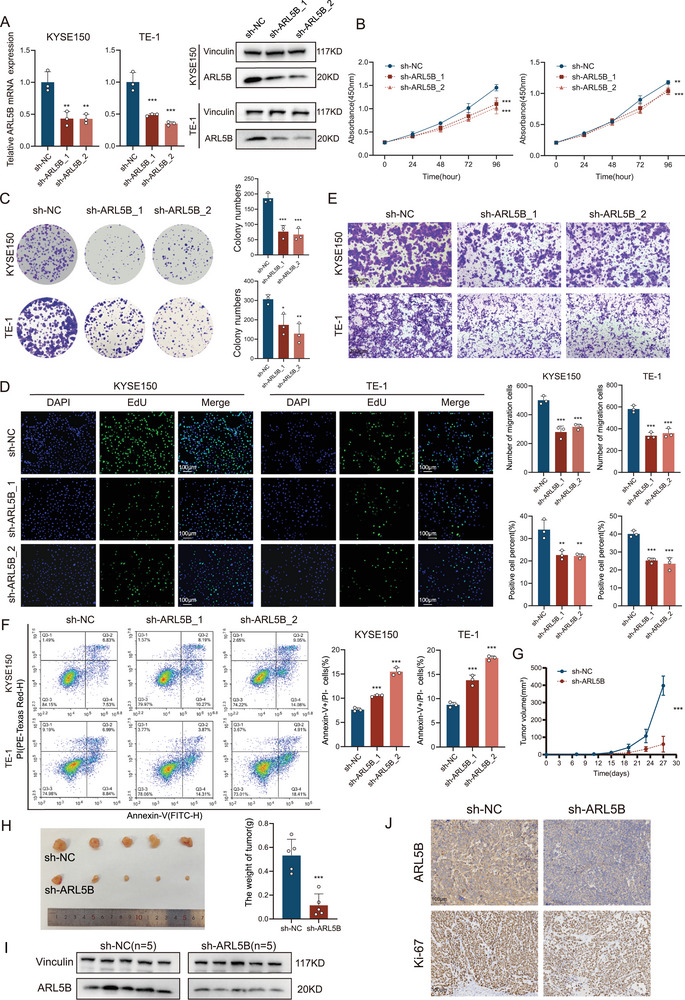
ARL5B promotes ESCC proliferation and migration in vitro and in vivo. A) Transfection efficiency was confirmed in KYSE150 and TE‐1 cells by qRT‐PCR and western blotting. B) CCK‐8 proliferation assays were performed in KYSE150 and TE‐1 cells with or without ARL5B knockdown (*n* = 4). C) Colony formation assay was performed to examine long‐term growth of KYSE150 and TE‐1 cells with or without ARL5B knockdown. D) The representative image of EdU incorporation assay in KYSE150 and TE‐1 cells with or without ARL5B knockdown, nuclei are counterstained with DAPI (blue). Right: Quantification of EdU‐positive cell percentage. Scale bars: 100 µm. E) Representative images and quantitative statistical data from Transwell invasion assays in KYSE150 and TE‐1 cells with or without ARL5B knockdown. F) Flow cytometry showed quantification of apoptotic cells (Annexin V‐positive) in KYSE150 and TE‐1 cells with or without ARL5B knockdown. G) Growth curves of subcutaneous xenograft tumors derived from KYSE150 cells expressing shNC or shARL5B_1 in BALB/c mice (*n* = 5 per group). H) Gross appearance and tumor weight of xenografts at study endpoint (day 27). I) Western blot analysis of ARL5B protein expression levels in xenograft tumor tissues. J) Representative immunohistochemical (IHC) staining images showing ARL5B and Ki67 expression in sections from xenograft tumors. Data are presented as mean SD. *, *p* < 0.05; **, *p* < 0.01; ***, *p* < 0.001; and ns, no significance.

Given ARL5B overexpression in ESCC, we next assessed its functional impact on cancer cell proliferation. ARL5B was knocked down in KYSE150 and TE‐1 cell lines using two distinct short hairpin RNA (shRNA) constructs (shARL5B_1/_2), qRT‐PCR and western blot analyses confirmed efficient suppression of ARL5B mRNA (KYSE150: *p* < 0.01, TE‐1: *p* < 0.001) and corresponding protein expression (both *p* < 0.05; **Figure** **2**A; Figure S1B, Supporting Information), followed by evaluation of phenotypes. We evaluated the impact of ARL5B depletion on ESCC growth kinetics. Cell counting kit‐8 (CCK‐8) assays revealed progressive suppression of cell viability in both shARL5B groups compared to controls, with the most pronounced inhibition observed at 96 h post seeding (both *p* < 0.001; Figure 2B). This inhibitory effect was corroborated by clonogenic assays, where ARL5B knockdown cells formed notably fewer and smaller colonies (KYSE150: *p* < 0.001; TE‐1: *p* < 0.05; Figure 2C). 5‐ethynyl‐2’‐deoxyuridine (EdU) incorporation assays were performed under normalized culture conditions. Quantitative image analysis demonstrated a sharp decrease in EdU‐positive nuclei (KYSE150: *p* < 0.01; TE‐1: *p* < 0.001; Figure 2D), unequivocally establishing ARL5B's pro‐proliferative function. Regarding invasive capacity, Transwell assay findings demonstrated that shRNA‐mediated ARL5B knockdown reduces cellular invasiveness in both KYSE150 and TE‐1 cell lines, demonstrating 40.4% and 40.1% reductions compared to scrambled controls, respectively (both *p* < 0.001; Figure 2E). Flow cytometric analysis of Annexin V/Propidium iodide (PI)‐stained cells demonstrated increased apoptosis rates in ARL5B‐depleted populations (both *p* < 0.001; Figure 2F).

To validate these phenotypic observations in a physiologically relevant context, we developed subcutaneous xenograft models. Lentiviral‐mediated ARL5B knockdown (shARL5B_1) in KYSE150 cells significantly attenuated tumor growth kinetics in BALB/c nude mice. By terminal sacrifice, tumors from the shARL5B group displayed significantly decreased tumor volume and mass compared to controls (both *p* < 0.001; Figure 2G,H), with ARL5B protein levels in tumor tissues confirming effective knockdown between the two groups (*p* < 0.05; Figure 2I; Figure S1C, Supporting Information).

Immunohistochemical analysis of harvested tumors confirmed sustained ARL5B suppression, and demonstrated concordant reduction in Ki67 proliferation index (Figure 2J; Figure S1D, Supporting Information), reinforcing the in vitro observations. These findings substantiate that ARL5B expression is instrumental for neoplastic progression in vitro cell culture systems and murine xenograft models.

### 2.3 ARL5B Promotes Tumor Progression through Fatty Acid Synthesis Mediated by SREBP1

**Figure 3:**
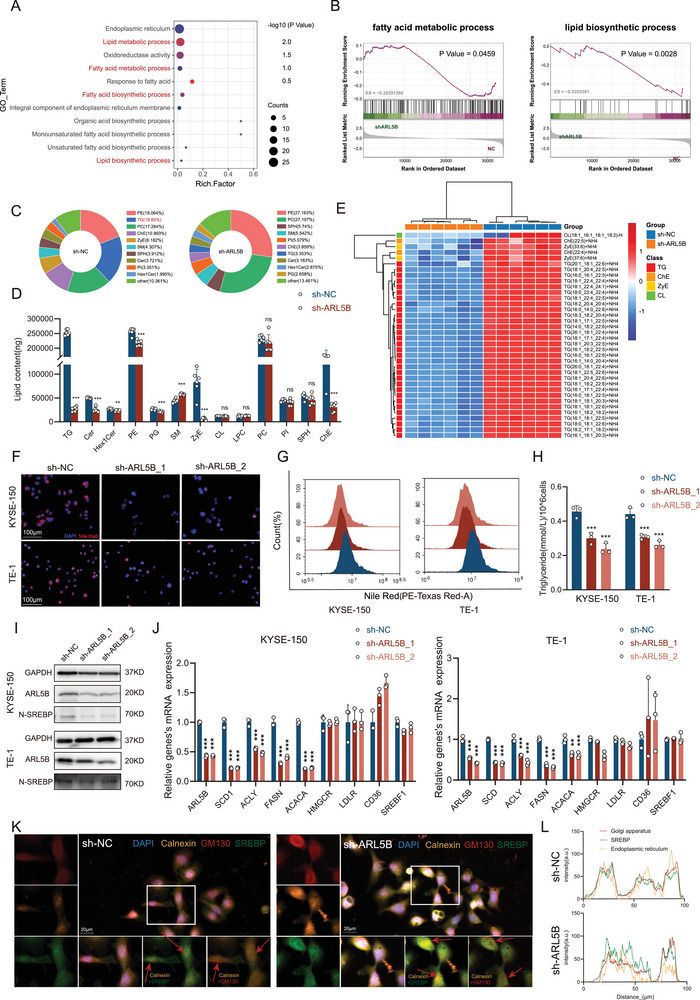
ARL5B promotes tumor progression through fatty acid synthesis mediated by SREBP1. A) Gene Ontology (GO) term enrichment analysis from RNA sequencing data of lipid metabolism‐related pathways KYSE150 cells with or without ARL5B knockdown. B) Gene set enrichment analysis (GSEA) plots depicting enrichment scores for the specific KEGG pathways “fatty acid metabolism process” and “lipid biosynthetic process”. C) Pie charts visualizing the overall composition of lipid classes identified by LC‐MS/MS lipidomic profiling in KYSE150 cells with or without ARL5B knockdown. D) Stacked bar graph shows quantified lipid classes based on analysis of LC‐MS/MS lipidomic profiling data (*n* = 6). E) Hierarchical clustering heatmap displaying the top 60 differentially regulated lipid species. F) Representative fluorescent images showing neutral lipid stained by Nile Red in KYSE150 and TE‐1 cells with or without ARL5B knockdown. Scale bars: 100 µm. G) Flow cytometry quantification plots of neutral lipids content in KYSE150 and TE‐1 cells with or without ARL5B knockdown. H) Cellular content of triglycerides was detected in KYSE150 and TE‐1 cells. I) Western blot analysis comparing the protein levels of N‐SREBP1 after ARL5B knockdown. J) qRT‐PCR quantification bar graphs comparing mRNA expression levels of key SREBP1 lipogenic target genes and genes involved in fatty acid uptake/degradation in KYSE150 and TE‐1 cells with or without ARL5B knockdown. K,L) Immunofluorescence analysis of SREBP subcellular localization in control and ARL5B knockdown cells, showing co‐localization with ER and Golgi apparatus. Scale bars: 20 µm. Data are presented as mean SD. *, *p* < 0.05; **, *p* < 0.01; ***, *p* < 0.001; and ns, no significance.

To systematically explore the downstream signaling pathways modulated by ARL5B, we performed RNA‐sequencing in KYSE150 cells with ARL5B knockdown. Volcano plot analysis identified 451 differentially expressed genes (DEGs, |log2FC| > 1, *p* < 0.05; Figure S1E, Supporting Information). Gene Ontology (GO) term analysis revealed profound enrichment of lipid metabolism‐related processes, particularly fatty acid biosynthesis (**Figure** **3**A). Concordantly, gene set enrichment analysis (GSEA) using Kyoto Encyclopedia of Genes and Genomes (KEGG) pathways demonstrated significant downregulation of “fatty acid metabolism” (*p* = 0.0459) and “lipid biosynthetic process” (*p* = 0.0028) in shARL5B cells, whereas fatty acid degradation, fat digestion and absorption, and fatty acid beta oxidation showed no significant alteration (Figure 3B; Figure S1F–I, Supporting Information). The plot illustrates differential expressed genes of lipid metabolism process (Figure S2B, Supporting Information). In contrast, GSEA revealed that metabolic pathways related to glucose, mitochondrial bioenergetics, and amino acid utilization were not significantly altered in ARL5B‐knockdown cells compared with controls (Figure S2A, Supporting Information). Functional validation by measuring cellular lactate levels and mitochondrial membrane potential confirmed that ARL5B knockdown did not cause significant changes in these pathways (Figure S2C,D, Supporting Information). These results largely ruled out alternative nonlipid metabolic routes in mediating the effects of ARL5B.

To functionally characterize the transcriptional changes, we performed comprehensive lipidomic profiling using Liquid Chromatography‐Tandem Mass Spectrometry (LC‐MS/MS) in control and ARL5B‐depleted cells. A total of 3590 lipid species spanning 47 classes were identified (Table S4, Supporting Information). Principal component analysis (PCA) revealed clear distinct clustering between groups (Figure S3A,B, Supporting Information), corroborated by pie chart visualization demonstrating qualitative compositional divergence (Figure 3C). Volcano plot analysis further identified 25 significantly downregulated lipid classes (|log2FC| > 1.5, *p* < 0.05; Figure S3C, Supporting Information), with triacylglycerols (TAGs) constituting the top ten most markedly reduced lipid ions (|log2FC| > 5, *p* < 0.05). Hierarchical clustering revealed TAGs as the most differentially regulated category. Quantitative analyses classified lipid classes into three groups: 25 downregulated, 17 stable, and 5 mildly upregulated (Figure 3D; Figure S3D, Supporting Information). Heatmap visualization confirmed a marked depletion of TAG species in ARL5B‐depleted cells (Figure 3E; Figure S2E, Supporting Information). These lipidomic alterations were consistent with the GSEA‐predicted suppression of lipid biosynthesis.

Functional validation in ESCC cell models further supported these findings. In ARL5B‐knockdown KYSE150 and TE‐1 cells, Nile‐Red‐based staining demonstrated diminished intracellular neutral lipid accumulation (both *p* < 0.001; Figure 3F; Figure S3F, Supporting Information), which was quantitatively confirmed by flow cytometric analysis (Figure 3G). Intracellular triglyceride measurements showed a significant reduction compared with controls (Figure 3H). Collectively, ARL5B orchestrates oncogenic adaptation by mediating metabolic reprogramming via regulation of fatty acid synthesis pathways.

Given the robust enrichment of lipid synthesis pathways, we focused on sterol regulatory element‐binding protein 1 (SREBP1), a canonical regulator of lipogenesis that drives de novo lipid biosynthesis through its active nuclear form (*N*‐SREBP1), which transcriptionally upregulates key enzymes including FASN, ATP‐citrate lyase (ACLY), SCD1, and acetyl‐CoA carboxylase alpha (ACACA). Western blot analysis showed that ARL5B knockdown markedly reduced nuclear SREBP1 levels (Figure 3I). Consistently, qRT‐PCR revealed significant downregulation of SREBP1 target genes (FASN, SCD1, ACLY, and ACACA), whereas genes involved in fatty acid uptake or oxidation (3‐hydroxy‐3‐methylglutaryl‐coenzyme A reductase, HMGCR; low‐density lipoprotein receptor, LDLR; CD36 molecule, CD36) remained unaffected (Figure 3J). This evidence from transcriptional profiling and target gene validation exhibits ARL5B as a crucial upstream regulator of SREBP1‐mediated lipogenic programming in ESCC pathobiology.

To elucidate the mechanism underlying ARL5B‐mediated regulation of SREBP1, we examined its intracellular trafficking. SREBP1 actication requires coat protein II (COPII) vesicle‐mediated transport from the endoplasmic reticulum (ER) to the Golgi for proteolytic maturation and subsequent nuclear translocation.^[^ ^24^ ^]^ Immunofluorescence analyses using ER (calnexin) and Golgi (GM130) markers revealed that ARL5B deficiency led to pronounced ER accumulation of SREBP1, accompanied by reduced Golgi localization compared with controls (Figure 3K,L). These results demonstrate that ARL5B knockdown impaired anterograde trafficking of SREBP1 from ER exit sites to *trans*‐Golgi networks, thereby attenuating its nuclear translocation.

### 2.4 Identification and Validation of the Direct Interaction between ARL5B and ROCK1

**Figure 4:**
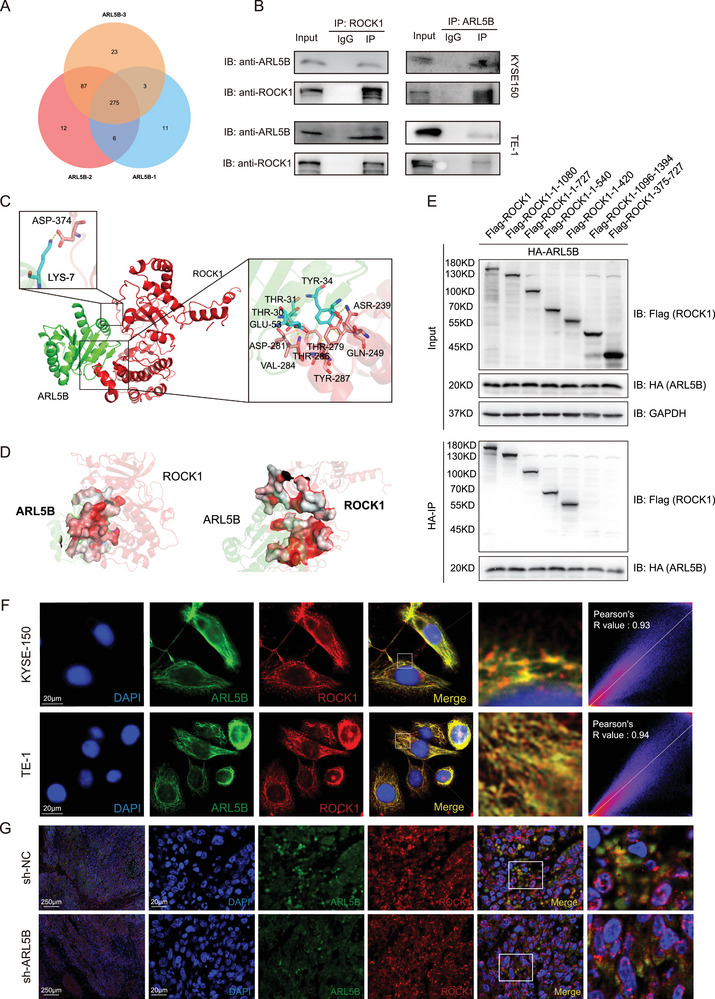
Identification and validation of the direct interaction between ARL5B and ROCK1. A) Venn diagram of ARL5B‐interacting proteins identified by Co‐IP coupled with LC‐MS/MS in KYSE150 (*n* = 3). B) Endogenous Co‐IP assays in KYSE150 and TE‐1 cells demonstrating reciprocal interaction between ARL5B and ROCK1. C) Protein–protein docking model showing the predicted binding interface of ARL5B (green) and ROCK1 (red). Key residues mediating intermolecular recognition are highlighted, including Thr‐30, Thr‐31, Tyr‐34, and Glu‐53 on ARL5B, and Asp‐281, Thr‐279, Thr‐286, and Asn‐239 on ROCK1. D) Hydrophobicity surface mapping of the ARL5B–ROCK1 interface. E) Co‐immunoprecipitation assays with wild‐type and six ROCK1 truncation mutants (ROCK1‐1‐1080, ROCK1‐1‐727, ROCK1‐1‐540, ROCK1‐1‐420, ROCK1‐1096‐1394, and ROCK1‐375‐727) co‐expressed with HA‐tagged ARL5B in 293T cells. F) Immunofluorescence analysis showing co‐localization of ARL5B (green) and ROCK1 (red) in KYSE150 and TE‐1 cells. Scale bars: 20 µm. G) Immunofluorescence co‐localization of ARL5B and ROCK1 in tumor sections from xenografts. Scale bars: left: 250 µm and right: 20 µm. Data are presented as mean SD. *, *p* < 0.05; **, *p* < 0.01; ***, *p* < 0.001; and ns, no significance.

To uncover molecular partners that may mediate the regulatory role of ARL5B, we performed co‐immunoprecipitation (Co‐IP) combined with LC‐MS/MS analysis in KYSE150 and TE‐1 cells. This analysis identified 417 ARL5B‐interacting candidates (**Figure** **4**A). Among these proteins, we prioritized those functionally implicated in COPII vesicle‐mediated ER‐to‐Golgi trafficking, including ROCK1, PI4KB, HSPA5, RAB10, and RAB1A. To further validate these candidates, endogenous Co‐IP assays were conducted in KYSE150 and TE‐1 cells. Immunoprecipitation of ARL5B successfully pulled down ROCK1, and reciprocal Co‐IP of ROCK1 confirmed this interaction (Figure 4B). By contrast, no interaction was observed between ARL5B and the other four candidates (Figure S4A, Supporting Information), indicating that ROCK1 is the predominant interactor of ARL5B in this context.

To obtain structural insights into the ARL5B–ROCK1 interaction, we performed protein–protein docking analysis. The docking model identified a stable interface where ARL5B engages ROCK1 through complementary structural motifs. Specifically, several amino acid residues at the interface were highlighted, including Thr‐30, Thr‐31, Tyr‐34, and Glu‐53 on ARL5B, and Asp‐281, Thr‐279, Thr‐286, and Asn‐239 on ROCK1, suggesting that these residues mediate direct intermolecular recognition (Figure 4C). In addition, surface hydrophobicity mapping further characterized the physicochemical nature of the interface. The ARL5B binding surface exhibited concentrated red hydrophobic patches, while the complementary ROCK1 surface also displayed extensive hydrophobic clusters (Figure 4D). The spatial alignment of these hydrophobic regions strongly supports that hydrophobic contacts represent a key driving force stabilizing ARL5B–ROCK1 binding.

To experimentally validate these predictions, we designed and constructed six ROCK1 truncation mutants (ROCK1‐1‐1080, ROCK1‐1‐727, ROCK1‐1‐540, ROCK1‐1‐420, ROCK1‐1096‐1394, and ROCK1‐375‐727). Wild‐type and truncated ROCK1 plasmids carrying a FLAG (DYKDDDDK) tag, together with an HA (hemagglutinin, YPYDVPDYA)‐tagged ARL5B plasmid, were transfected into 293T cells, and Co‐IP assays were subsequently performed to define the domains required for their interaction. The Co‐IP results revealed that the 1–420 domain of ROCK1 is the major region responsible for binding to ARL5B (Figure 4E), which is consistent with the molecular docking results. Taken together, these analyses provide mechanistic evidence supporting the direct interaction between ARL5B and ROCK1.

We next investigated the spatial distribution of ARL5B and ROCK1 within cells. Immunofluorescence staining in KYSE150 and TE‐1 cells revealed striking co‐localization of ARL5B and ROCK1, with Pearson's correlation coefficients of 0.93 and 0.94, respectively (Figure 4F; Figure S4B, upper, Supporting Information). This co‐localization pattern was also observed in tumor xenograft sections, where ARL5B overexpression further enhanced the overlap between ARL5B and ROCK1 signals (Figure 4G; Figure S4B (lower),S4C, Supporting Information). These findings provide in situ confirmation of their physical association and underscore the biological relevance of ARL5B–ROCK1 interaction in ESCC.

### 2.5 ROCK1 Is Required for ARL5B‐Induced Lipid Metabolic Reprogramming

**Figure 5:**
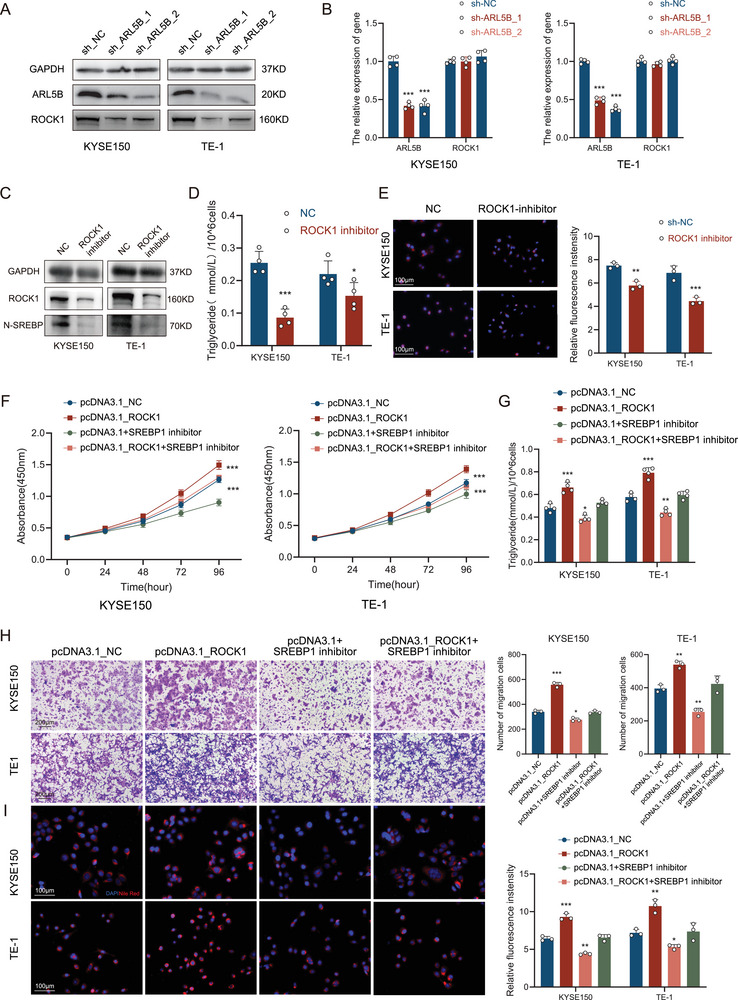
ROCK1 is required for ARL5B‐induced lipid metabolic reprogramming. A) Western blot analysis of ARL5B and ROCK1 protein expression in NC and ARL5B‐knockdown KYSE150 and TE‐1 cells. B) Relative mRNA expression levels of ARL5B and ROCK1 in NC and ARL5B‐knockdown KYSE150 and TE‐1 cells. C) Immunoblot analysis of N‐SREBP1 levels in KYSE150 and TE‐1 cells treated with 5 µm ROCK1 inhibitor TLC2976‐0103 for 48 h. D) Quantification of intracellular triglyceride content in KYSE150 and TE‐1 cells following 48 h treatment with 5 µm TLC2976‐0103. E) Representative fluorescent images and quantification of neutral lipid accumulation stained with Nile Red in KYSE150 and TE‐1 cells after 48 h treatment with 5 µm TLC2976‐0103. F) CCK‐8 assays were performed in control and ROCK1‐overexpressing KYSE150 and TE‐1 cells treated ±10 µm fatostatin hydrobromide for 48 h. G) Quantification of intracellular triglyceride content in control and ROCK1‐overexpressing KYSE150 and TE‐1 cells with or without fatostatin hydrobromide. H) Migration ability was assessed by Transwell assays in control and ROCK1‐overexpressing KYSE150 and TE‐1 cells exposed to fatostatin hydrobromide or vehicle. I) Nile Red staining and fluorescence quantification showing lipid droplet accumulation in control and ROCK1‐overexpressing KYSE150 and TE‐1 cells in the presence or absence of fatostatin hydrobromide.

We next examined whether ARL5B regulates ROCK1 expression. Western blot analysis demonstrated that ROCK1 protein levels were upregulated upon ARL5B overexpression and downregulated following ARL5B knockdown as shown by representative immunoblots and corresponding grayscale quantification (**Figure** **5**A; Figure S4D,E, Supporting Information), whereas ROCK1 mRNA levels remained unchanged (Figure 5B; Figure S4E, Supporting Information). These findings indicate that ARL5B modulates ROCK1 protein expression in a post‐transcriptional manner, thereby influencing *N*‐SREBP1 activation. To validate the regulatory role of ROCK1 in SREBP1 activation, we treated KYSE150 and TE‐1 cells with a ROCK1‐specific inhibitor TLC2976‐0103. Immunoblot analysis demonstrated a significant reduction in N‐SREBP1 levels following 48 h treatment of KYSE150 and TE‐1 cells with 5 µm TLC2976‐0103 (Figure 5C; Figure S4F, Supporting Information; both *p* < 0.05), accompanied by coordinated decreases in intracellular triglyceride accumulation (KYSE150: *p* < 0.001; TE‐1: *p* < 0.05; Figure 5D) and lipid droplet quantity (KYSE150: *p* < 0.01; TE‐1: *p* < 0.001; Figure 5E), which is consistent with the inhibition of SREBP1‐mediated lipogenic activity. These findings establish ROCK1 as a critical ARL5B interactor and mediator of SREBP1 processing, mechanistically bridging ARL5B's regulation of vesicular trafficking to lipid metabolic reprogramming in ESCC.

To provide functional confirmation, rescue experiments were conducted by combining ROCK1 overexpression with pharmacological blockade of SREBP1. KYSE150 and TE‐1 cells were transfected with ROCK1 overexpression plasmids and subsequently treated with the SREBP1 inhibitor fatostatin hydrobromide (10 µm, 48 h). CCK‐8 assays revealed that ROCK1 overexpression significantly enhanced cell proliferation, whereas fatostatin hydrobromide treatment abrogated this effect (both *p* < 0.001; Figure 5F). Consistently, Transwell migration assays demonstrated that ROCK1 overexpression promoted cell migration, while fatostatin hydrobromide markedly suppressed this phenotype, with quantitative analysis of migrated cell counts confirming statistical significance (KYSE150: *p* < 0.05; TE‐1: *p* < 0.01; Figure 5H). In parallel, lipid metabolic changes were assessed. Nile Red staining combined with fluorescence quantification showed that ROCK1 overexpression markedly increased intracellular lipid droplet accumulation, whereas fatostatin hydrobromide treatment reduced both the number and fluorescence intensity of lipid droplets (KYSE150: *p* < 0.01; TE‐1: *p* < 0.05; Figure 5I). Triglyceride assays further confirmed a significant elevation of triglyceride levels in ROCK1‐overexpressing (OE) cells, which was effectively reversed upon fatostatin hydrobromide treatment (KYSE150: *p* < 0.05; TE‐1: *p* < 0.01; Figure 5G). These findings demonstrate that ROCK1 is indispensable for ARL5B‐induced lipid metabolic reprogramming. Moreover, the rescue experiments establish that the ARL5B–ROCK1 axis promotes tumor progression via SREBP1‐mediated lipogenesis, highlighting the functional significance of the ARL5B–ROCK1–SREBP1 cascade in ESCC.

### 2.6 The ARL5B–ROCK1–SREBP1 Axis Drives Lipogenesis and Malignancy

**Figure 6:**
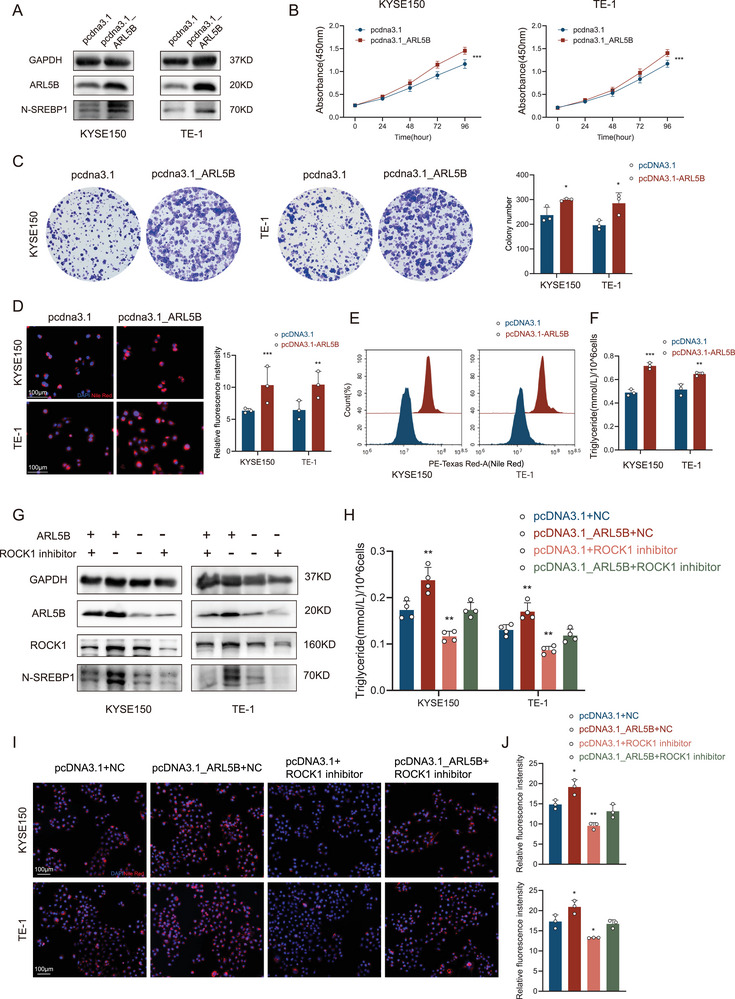
The ARL5B‐ROCK1‐SREBP1 axis drives lipogenesis and malignancy. A) Immunoblot analysis of N‐SREBP1 levels in control and ARL5B‐overexpressing KYSE150 and TE‐1 cells. B) CCK‐8 proliferation assays in control and ARL5B‐overexpressing KYSE150 and TE‐1 cells. C) Colony formation assays in control and ARL5B‐overexpressing KYSE150 and TE‐1 cells. D) Nile Red staining of intracellular neutral lipids in control and ARL5B‐overexpressing KYSE150 and TE‐1 cells, quantified by relative fluorescence intensity. E) Flow cytometry quantification of Nile Red fluorescence intensity in control and ARL5B‐overexpressing KYSE150 and TE‐1 cells. F) Quantification of intracellular triglyceride levels in control and ARL5B‐overexpressing KYSE150 and TE‐1 cells. G) Immunoblot analysis of N‐SREBP1 levels in control and ARL5B‐overexpressing cells with or without 5 µm TLC2976‐0103 treatment. H) Cellular content of triglycerides in control and ARL5B‐overexpressing cells with or without 5 µm TLC2976‐0103 treatment. I,J) Nile Red staining of intracellular neutral lipids in control and ARL5B‐overexpressing cells with or without 5 µm TLC2976‐0103 treatment, quantified by relative fluorescence intensity. Scale bars: 100 µm. Data are presented as mean SD. *, *p* < 0.05; **, *p* < 0.01; ***, *p* < 0.001; and ns, no significance.

To further delineate the functional role of the ARL5B–ROCK1 axis in lipid metabolic reprogramming, we overexpressed ARL5B in KYSE150 and TE‐1 cells and evaluated its oncogenic effects. Consistent with our previous findings, western blot analysis revealed that ARL5B overexpression significantly upregulated the active N‐SREBP1 (**Figure** **6**A), a master transcription factor governing lipid biosynthesis. Functional assays performed in KYSE150 and TE‐1 demonstrated that ARL5B‐overexpressing cells exhibited enhanced proliferative capacity, as evidenced by increased CCK‐8 absorbance (both *p* < 0.001; Figure 6B) and elevated colony formation (both *p* < 0.05; each well seeded with 2000 cells; Figure 6C) compared to control groups. To further validate the involvement of ARL5B in oncogenic lipid metabolic reprogramming, we analyzed lipid accumulation by Nile Red staining. ARL5B‐overexpressing cells displayed a striking increase in intracellular lipid droplets (KYSE150: *p* < 0.001; KYSE150: *p* < 0.01; Figure 6D), a result corroborated by flow‐cytometry‐based quantification of Nile Red fluorescence intensity (Figure 6E). ARL5B overexpression substantially elevated cellular triglyceride content in both cell lines (KYSE150: *p* < 0.001; TE‐1: *p* < 0.01; Figure 6F). To validate the dependency of these effects on ROCK1, rescue experiments were performed using the selective ROCK1 inhibitor (TLC2976‐0103). Treatment with 5 µm TLC2976‐0103 for 48 h in ARL5B‐overexpressing cells significantly attenuated N‐SREBP1 protein levels (Figure 6G), indicating that ARL5B regulates SREBP1 maturation through ROCK1. Moreover, ROCK1 inhibition reversed lipid accumulation induced by ARL5B overexpression, as shown by reduced triglyceride content (both *p* < 0.01; Figure 6H), diminished lipid droplets, and decreased Nile Red fluorescence intensity (both *p* < 0.05, Figure 6I,J). Importantly, these results aligned with our earlier observation that ROCK1 knockdown suppresses ESCC progression, this finding is also supported by previous studies,^[^ ^25^, ^26^ ^]^ thereby further solidifying ROCK1 as a downstream effector of ARL5B in lipid metabolic reprogramming. Collectively, these data reveal that ARL5B drives lipid synthesis and tumor progression via ROCK1‐mediated activation of SREBP1, providing mechanistic insights into how metabolic alterations fuel ESCC aggressiveness.

### 2.7 Pharmacological Inhibition of SREBP1 Abrogates ARL5B‐Driven Oncogenic Effects In Vitro and In Vivo

**Figure 7:**
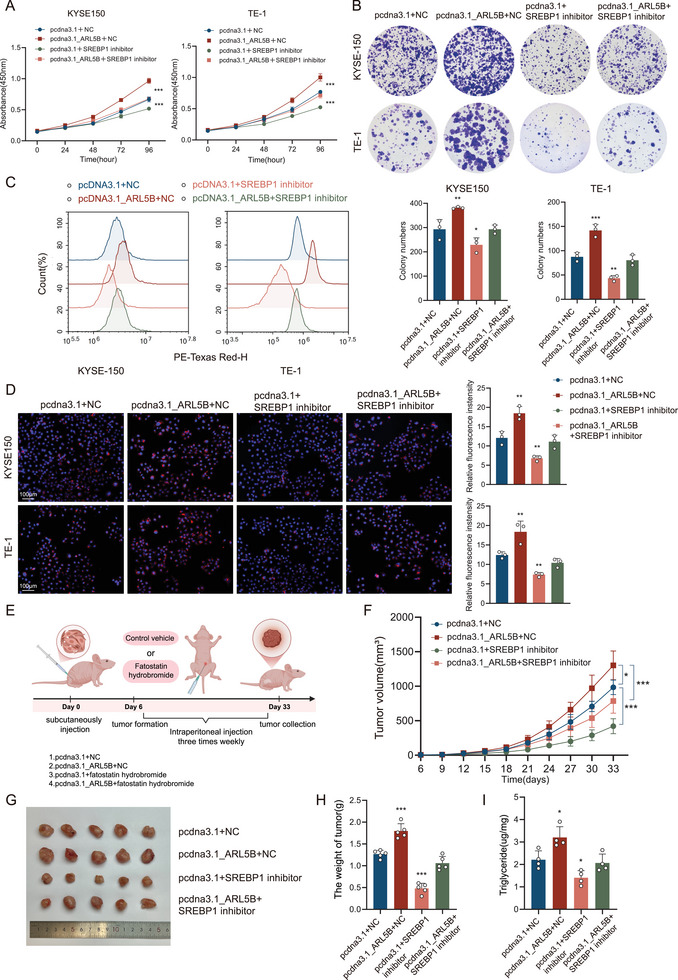
Pharmacological inhibition of SREBP1 abrogates ARL5B‐driven oncogenic effects in vitro and in vivo. A) CCK‐8 proliferation assays were performed in KYSE150 and TE‐1 cells with ARL5B overexpression or 10 µm fatostatin hydrobromide treatment (*n* = 4). B) Colony formation assay was performed in KYSE150 and TE‐1 cells with ARL5B overexpression or 10 µm fatostatin hydrobromide treatment; lower, quantification of colony number. C) Flow cytometry quantification of Nile Red fluorescence intensity in KYSE150 and TE‐1 cells with indicated treatment. D) Detection of neutral lipids by Nile Red staining in KYSE150 and TE‐1 cells with indicated treatment. Right, quantification of fluorescence intensity with ImageJ. Scale bar: 100 µm. E) Schematic of the xenograft study design: BALB/C mice (*n* = 5 per group) subcutaneously injected with ARL5B‐OE or NC KYSE150 cells, from day 6, mice received intraperitoneal injections of either fatostatin hydrobromide (25 mg kg^−1^, dissolved in dimethyl sulfoxide (DMSO) and diluted in PBS) or the corresponding vehicle control (PBS with equal DMSO concentration) three times weekly until day 33 post inoculation. F) Growth curves of subcutaneous xenografts. G) Gross appearance of subcutaneous xenografts at study endpoint. H) Quantitative analysis of xenograft weight. I) Detection of triglyceride content in harvested xenograft tumors. Data are presented as mean SD. *, *p* < 0.05; **, *p* < 0.01; ***, *p* < 0.001; and ns, no significance.

To functionally interrogate the necessity of SREBP1 in ARL5B‐mediated tumor progression, rescue experiments were conducted fatostatin hydrobromide in both cellular and xenograft models. Building upon our findings that ARL5B activates SREBP1 to promote lipid reprogramming, we established four experimental groups: ARL5B‐OE cells with/without fatostatin hydrobromide treatment, and negative control (NC) cells with/without fatostatin hydrobromide treatment. CCK‐8 and colony formation assays demonstrated that ARL5B‐OE significantly enhanced KYSE150 and TE‐1 cell proliferation (both *p* < 0.001, **Figure** **7**A; both *p* < 0.01, Figure 7B), whereas 48 h pharmacological exposure to 10 µm fatostatin hydrobromide elicited comparable antiproliferative effects in both NC controls and ARL5B‐OE cohorts (both *p* < 0.001, Figure 7A; both *p* < 0.001, Figure 7B). Coherent with our prior lipidomic analyses, Nile red staining coupled with quantitative flow cytometry revealed that ARL5B‐OE cells exhibited increased lipid droplet accumulation, which was markedly diminished by fatostatin hydrobromide (both *p* < 0.01; Figure 7C,D). These data underscore that SREBP1 activation is indispensable for ARL5B‐induced lipogenesis and proliferative gains. To validate these findings in xenograft models, ARL5B‐OE or NC KYSE150 cells were subcutaneously injected into female BALB/c nude mice (*n* = 5). Following tumor establishment, mice were administered intraperitoneal phosphate buffered saline (PBS) or fatostatin hydrobromide (25 mg kg^−1^) three times weekly until day 33 post‐inoculation (schematized in Figure 7E). Consistently, ARL5B‐OE tumors exhibited significantly greater volume (*p* < 0.05) and mass (*p* < 0.001) compared to the NC group. In contrast, fatostatin hydrobromide treatment substantially suppressed tumor growth in ARL5B‐OE xenografts (*p* < 0.001; Figure 7F–H). Biochemical analysis of explanted tumors demonstrated parallel trends in triglyceride content, with ARL5B‐OE tumors exhibiting higher levels than NC controls (*p* < 0.05), which were reduced by following fatostatin hydrobromide administration (*p* < 0.05; Figure 7I). These experiments emphasizing the axis‐specific dependency on SREBP1 for lipid‐driven oncogenesis. Taken together, these rescue experiments provide validation that the ARL5B–ROCK1–SREBP1 axis constitutes a druggable metabolic vulnerability in ESCC. The reversal of both lipid accumulation and tumor growth upon SREBP1 inhibition solidifies its role as the critical downstream effector mediating ARL5B's oncogenic functions.

## 3 Conclusion 

**Figure 8:**
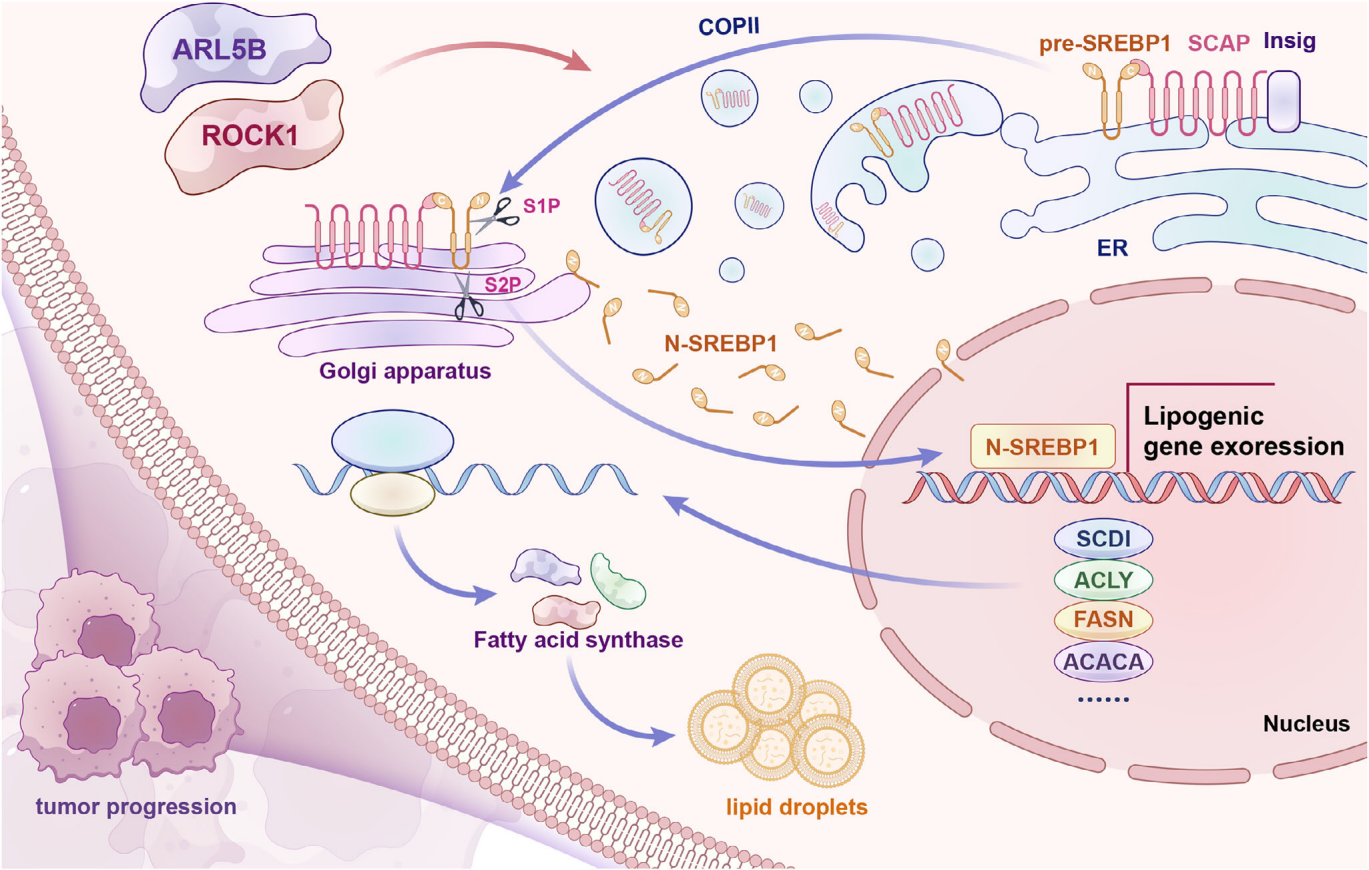
ARL5B drives esophageal squamous cell carcinoma progression via ROCK1–SREBP1‐mediated lipid metabolic reprogramming. S1P and S2P: site‐1 proteases and site‐2 proteases. N‐SREBP1: nuclear SREBP1.

We have for the first time elucidated the molecular mechanism (**Figure** **8**) by which ARL5B drives lipid metabolic reprogramming and malignant progression in ESCC through regulating the ROCK1‐mediated SREBP1 activation pathway. ARL5B overexpression serves as an independent prognostic risk factor for poor outcomes in ESCC patients. Targeting the ARL5B–ROCK1–SREBP1 axis may represent a novel strategy to counteract metabolic dysregulation and treat ESCC. This study establishes a crucial theoretical foundation for molecular subtyping and precision therapy of ESCC.

## 4 Discussion

ESCC remains a highly aggressive gastrointestinal cancer with persistently poor outcomes despite advances in basic research and clinical research, highlighting underscoring the need for precise therapeutic targets.^[^ ^29^ ^]^ Here, we identify the first evidence that ARL5B serves as a key driver of ESCC progression via the ARL5B/ROCK1/SREBP1 signaling axis. These findings offer novel insights into controlling ESCC malignancy and suggest that ARL5B may represent a promising ESCC‐specific therapeutic target with translational potential.

As a member of the ARF family, which regulates tumorigenesis across multiple cancer types, ARL5B is identified here as an oncogenic driver in ESCC. Interrogation of GEO datasets revealed significant ARL5B upregulation in ESCC tissues. Clinical correlation further demonstrated that elevated ARL5B expression correlated with advanced tumor progression and inferior survival, supporting a functional role in ESCC pathogenesis. Mechanistically, ARL5B overexpression robustly activated fatty acid metabolic pathways, particularly those involving key enzymes such as SCD1, ACLY, and FASN, confirming its role in fueling ESCC malignancy through lipid biosynthesis.

Given that our study specifically targets fatty acid synthesis, we focused on SREBP1—the master transcriptional switch of this program. SREBPs comprise two isoforms: SREBP1, which controls fatty acid and triglyceride synthesis, and SREBP2, which governs cholesterol metabolism.^[^ ^23^ ^]^ SREBP1 has been implicated in tumor progression across multiple cancers, including ESCC,^[^ ^30^, ^31^, ^32^, ^33^, ^34^, ^35^ ^]^ where it promotes epithelial–mesenchymal transition, Wnt/β‐catenin activation, and radiotherapy resistance via SCD1‐mediated lipid remodeling.^[^ ^21^ ^]^ Recent studies, such as those by Shen and co‐workers, highlight how PRP19 stabilizes SREBF1 mRNA through m6A‐dependent mechanisms to drive ESCC progression.^[^ ^36^ ^]^ Pharmacological SREBP1 inhibitors, including fatostatin and betulin, have shown potent preclinical antitumor efficacy.^[^ ^30^, ^33^, ^37^, ^38^ ^]^ Consistent with these observations, in our study SREBP1 inhibition curtailed ESCC growth in vitro and in vivo and partially abrogated ARL5B‐driven oncogenic effects.

We next investigated whether ARL5B activates SREBP1. Enforced ARL5B expression markedly increased the protein abundance of nuclear SREBP1 without altering SREBP1 transcript levels, indicating post‐transcriptional control. Because SREBP1 activation requires COPII vesicle‐mediated trafficking from the ER to the Golgi followed by site‐1/site‐2 proteolysis,^[^ ^39^, ^40^, ^41^ ^]^ we assessed transport dynamics by immunofluorescence. ARL5B knockdown impaired COPII‐dependent ER‐to‐Golgi trafficking of SREBP1 precursors and thereby prevented their maturation, revealing a previously unrecognized mechanism for SREBP1 activation. Future studies should focus on real‐time visualization of SREBP1 vesicular trafficking and nuclear translocation.

We have screened out candidate molecules (ROCK1, HSPA5, PIK4B, RAB1A, and RAB10) regulating COPII vesicle transport through proteomic profiling of ARL5B‐interacting proteins. ROCK1, which is a key kinase governing cytoskeletal dynamics, cell motility, and tumor microenvironment remodeling,^[^ ^26^ ^]^ has been implicated in COPII vesicle biogenesis through macrolevel regulation of SEC23A (SEC23 Homolog A, COPII Coat Complex Component) expression.^[^ ^42^ ^]^ Accumulating evidence implicates ROCK1 in diverse malignancies—including ESCC, prostate and gastric cancers, and osteosarcoma.^[^ ^43^, ^44^, ^45^ ^]^ Here, we show that ROCK1 engages a COPII‐dependent trafficking route to activate SREBP1, thereby rewiring lipogenic programmes and promoting ESCC progression. Pharmacological ROCK1 inhibition (for example, with fasudil or Y‐27632) reduced N‐SREBP1 abundance, dampened lipid synthesis, and curtailed tumor growth in vitro and in vivo.^[^ ^46^ ^]^

This work identifies the ARL5B/ROCK1/SREBP1 axis as a novel signaling cascade promoting ESCC progression through lipid metabolic reprogramming. Importantly, we identify ROCK1 as a mediator of SREBP1 activation, extending the current understanding of SREBP1 regulatory mechanisms.^[^ ^47^ ^]^ Clinically, targeting ARL5B may achieve dual benefits: 1) ARL5B levels could guide personalized treatment and prognosis assessment in ESCC, and 2) combining existing ROCK1 inhibitors (e.g., fasudil) with SREBP1 inhibitors (e.g., fatostatin) may enhance therapeutic efficacy in ARL5B‐high tumors. Patient‐derived xenograft (PDX) models and preclinical trials are warranted to validate these hypotheses. Two limitations merit attention: current models incompletely recapitulate tumor microenvironment‐mediated lipid metabolism remodeling, and specific ARL5B inhibitors remain unavailable for therapeutic validation. Collectively, our findings position ARL5B as a critical node in ESCC pathogenesis by coupling ROCK1‐ to SREBP1‐dependent lipogenesis. These data nominate the ARL5B–ROCK1–SREBP1 axis as a tractable therapeutic vulnerability in ESCC.

## 5 Experimental Section

### Ethical Compliance and Tissue Specimen Acquisition

This study adhered to the guidelines of the Declaration of Helsinki and was approved by the Medical Ethics Committee of Shandong University School of Clinical Medicine (approval number: KYLL‐2021(KS)‐211). A total of 25 human esophageal tissue sections, including 5 cases each of normal esophageal epithelium and stage I–IV ESCC, were obtained from the Department of Pathology at Qilu Hospital of Shandong University between 1 January 2011 and 30 December 2014. Tumor staging was rigorously confirmed according to the Eigth edition of the American Joint Committee on Cancer (AJCC) guidelines by two independent pathologists. Additionally, 12 pairs of surgically resected ESCC tumors and paired adjacent normal tissues were collected from the Department of Thoracic Surgery at the same institution from 1 October 2023 to 1 January 2024, for subsequent protein extraction and western blot analysis. Clinical information for ESCC patients whose tissues were used for immunohistochemistry and western blotting is provided in Table S1 (Supporting Information).

### Bioinformatics Analysis

ESCC mRNA expression files were extracted from TCGA using Genomic Data Commons Data Portal (https://portal.gdc.cancer.gov/). Data included RNA‐seq data of 182 primary ESCA and 286 paired normal esophageal specimens. ESCA samples were first ranked based on the expression of ARL5B and the samples were divided into high ARL5B expression group and low ARL5B expression group. Further, paired RNA‐seq data of ESCC specimens were obtained from the GEO database under accession numbers GSE167488, GSE75241. GSEA was carried out using OmicShare tools (https://www.omicshare.com/ tools). In addition, online visualization sites including GEPIA (https://gepia.cancer‐pku.cn/), UALCAN (https://ualcan.path.uab.edu/), and ENCORI (https://starbase.sysu.edu.cn/) were used to analyze mRNA expression and common pathways of ARL5B in various tumors.

### Immunohistochemistry

After deparaffinization, rehydration, and antigen retrieval, slides were incubated with primary rabbit antihuman ARL5B (dilution 1:100; Cusabio technology Antibody; #CSB‐PA002091GA01HU) overnight at 4 °C. The slides were then incubated with antirabbit secondary antibody followed by chromogen diaminobenzidine (DAB) staining and hematoxylin counterstaining, and mounted with xylene‐based medium. The tissue slides were quantitatively scored under a microscope according to the percentage of positive cells and staining intensity. An *H*‐score (maximum score, 300) was assigned using the following formula: 3 × percentage of strongly staining nuclei + 2 × percentage of moderately staining nuclei + percentage of weakly staining nuclei, giving a range of 0–300.^[^ ^27^ ^]^ Two experienced pathologists who were blinded to the clinical information independently validated the reproducibility of the scoring system.

### Cell Culture

The HEK293T cells and normal esophageal squamous cell line Het‐1A cells (CCTCC, Wuhan, China) were cultured in dulbecco's modified eagle medium (DMEM) (Gibco, Life Technologies Inc., Grand Island, NY, USA), and human ESCC cells TE‐1, KYSE150, ECA109, and KYSE510 (CCTCC, Wuhan, China) were cultured in RPMI 1640 media (Gibco). The media were supplemented with 10% fetal bovine serum (FBS) (Gibco) and 1% antibiotics (Gibco) were added to the medium. All cells were maintained at 37 °C in a humidified chamber with 5% CO_2_.

### RNA Extraction and Quantitative qRT‐PCR Analysis

TRIzol (Invitrogen, USA) was used to extract total RNA according to manufacturer's instruction. The high‐capacity complementary deoxyribonucleic acid (cDNA) Reverse Transcription Kit (Yeason, China) was leveraged for reverse transcription in accordance with the manufacturer's protocol. A quantitative PCR system (Roche, Switzerland) was used for qRT‐PCR. The primers of glyceraldehyde‐3‐phosphate dehydrogenase (GAPDH) and ARL5B used for qRT‐PCR were 5′‐GCACCGTCAAGGCTGAGAAC‐3′ (forward) and 5′‐TGGTGAAGACGCCAGTGGA‐3′ (reverse) for GAPDH; and 5′‐AGTGGGACTGGATAATGCAGGG‐3′ (forward) and 5′‐ATCGCAGAGACTCCTGACCACC‐3′ (reverse) for ARL5B. Primers for other genes are listed in Table S2 (Supporting Information).

### Plasmids and Lentiviruses

Lentivirus‐expressing shRNAs targeting ARL5B (sh‐ARL5B_1, S: CCACCAUUCUUUACCAAUUTT; AS: AAUUGGUAAAGAAUGGUGGTT; sh‐ARL5B_2, S: CAUAGGAAGCAAUGUUGAAGA; AS: UUCAACAUUGCUUCCUAUGGU) were synthesized by Shanghai GeneChem Co., Ltd. (Shanghai, China), and were added to KYSE150 and TE‐1 to knockdown ARL5B. KYSE150 and TE‐1 were cultured for 72 h following infection and selected in puromycin (2 µg mL^−1^). Effective knockdown was evaluated by western blot analysis.

### CCK‐8 Assay and EdU Assay

CCK‐8 reagent (Yeason, 40203ES60, China) was used to assess sh‐ARL5B‐KYSE150 cells or sh‐Control‐KYSE150 cells viability. The sh‐ARL5B‐KYSE150 cells or sh‐Control‐KYSE150 were seeded in 96‐well plates at a density of 2.5 × 10^3^ cells per well in 100 µL of Gibco 1640 containing 10% FBS. After incubation for 24, 48, 72, and 96 h, 10 µL of CCK‐8 solution was added to each well for 1 h before measurement. Absorbance (optical density, OD value) at 450 nm was measured using a microplate reader (TECAN, Switzerland). EdU cell proliferation assay kit (Yeason, 40275ES60, China) was used to determine cell proliferation. Cells were incubated with 200 µL of 5‐ethynyl‐20‐deoxyuridine at 37 °C for 2 h. After fixed and permeabilized, the cells were incubated with Apollo reagent for 30 min and the Hoechst were used to stain nuclei. The images were viewed and obtained using a fluorescence microscope (Echo Revolve, San Diego, CA, USA).

### Colony Formation Assay

1000–2000 ESCC cells were seeded in 6‐well plates per well in 1.5 mL of Gibco 1640 containing 10% FBS. The cells were incubated in a chamber containing 5% CO_2_ and 5% O_2_ at 37 °C for 2 weeks. And colonies were fixed and stained with crystal violet (Solarbio, China) for 20 min. The colonies were washed with PBS for at least three times and the number of colonies were counted and analyzed.

### Cell Migration Assay

Cell migration assays were performed using Transwell chambers (8 µm pore size; Cat. No. 3422; Corning, USA). For migration assays, 5 × 10⁴ cells in 200 µL of serum‐free medium were seeded into the upper chamber, while 600 µL of medium containing 30% FBS was added to the lower chamber as a chemoattractant. After incubation for 48 h at 37 °C, nonmigrated or noninvaded cells remaining on the upper surface of the membrane were gently removed with a cotton swab. Cells that had migrated or invaded to the lower surface were fixed with 4% paraformaldehyde for 20 min, stained with 0.1% crystal violet, and counted under a light microscope in at least five randomly selected fields.

### Apoptosis Assay

An fluorescein isothiocyanate (FITC)–Annexin V apoptosis detection kit (BestBio, BB‐4101, China) was used according to the manufacturer's protocol to determine the proportion of apoptotic cells. In brief, treated cells were stained with Annexin V and PI solution in the dark for 15 min, and then analyzed by flow cytometry (Agilent NovoCyte Advanteon, USA). The data were further processed with FlowJo V10(Beckton Dickenson).

### Western Blot Analysis

Total protein was extracted from the subcutaneous tumor of mice using the total protein extraction kit (BC3710, Solarbio, China). Protein lysates were extracted from cultured cells using radioimmunoprecipitation assay buffer (RIPA) lysis buffer (R0010, Solarbio, China) supplemented with a protease inhibitor cocktail (CWbio, China). Equal amounts of protein samples were separated on 10–12.5% sodium dodecyl sulfate –polyacrylamide gel electrophoresis (SDS–PAGE) and transferred to polyvinylidene difluoride (PVDF) membranes (Millipore, USA). The membranes were blocked with 5% skim milk for 1 h at room temperature and incubated with primary antibodies (Table S3, Supporting Information) at 4 °C overnight. On the next day, the membranes were incubated with peroxidase‐conjugated secondary antibodies (1:5000, Cell Signaling, 7074, 7076, USA) for 1 h at room temperature, followed by detection using a chemiluminescent substrate (Millipore, USA) and chemiluminescent instrument (Tonon 5200, China). Images were analyzed with ImageJ software (Media Cybernetics Inc., USA). The primary antibodies used are listed in Table S3 (Supporting Information).

### RNA‐seq Analysis

ARL5B‐knockdown and control KYSE150 cells were cultured, and three independent samples were collected by Trizol reagent (Invitrogen, Carlsbad, CA, USA) and subjected to mRNA‐seq by the LC‐Bio (Zhejiang, China) and data analysis. Venn, GO enrichment, and KEGG enrichment analyses were performed as described by LC‐Bio (https://www.lc‐bio.cn/).

### Lipidomics

Lipids were extracted using the methyl tertiary butyl ether (MTBE) method. An aliquot of tissue was homogenized with 200 µL deionized water and 20 µL internal lipid standard mixture. The sample was vortexed, followed by sequential addition of 800 µL MTBE and 240 µL methanol precooled, with 1 min vortexing after each step. The mixture was ultrasonicated in an ice‐water bath for 20 min, equilibrated at 25 °C for 30 min, and centrifuged at 14 000 × *g* and 10 °C for 15 min. The organic phase was collected, dried under nitrogen, and reconstituted in 200 µL of 90% isopropanol/acetonitrile. After thorough vortexing, 90 µL of the solution was centrifuged at 14 000 × *g* and 10 °C for 15 min, and the supernatant was collected for LC‐MS analysis. Lipid analysis by LC‐MS/MS was performed using a Q‐Exactive Plus mass spectrometer (Thermo Scientific) coupled with a Ultra‐High Performance Liquid Chromatography (UHPLC) Nexera LC‐30A system (SHIMADZU). Separation was achieved using ACQUITY Ultra Performance Liquid Chromatography (UPLC) CSH C18 column (1.7 µm, 2.1 mm × 100 mm, Waters) with a mobile phase flow rate of 0.3 mL min^−1^ and an injection volume of 3 µL.The gradient elution program employed two phases: solvent A consisting of acetonitrile–water (6:4, v/v) with 0.1% formic acid and 0.1 mm ammonium formate, and solvent B containing acetonitrile–isopropanol (1:9, v/v) with 0.1% formic acid and 0.1 mm ammonium formate. The gradient program conditions were as follows: 0–2 min, 30% B; 2–25 min, 30–100% B; 25–26 min, 100–30% B; 26–35 min, 30% B. Samples were maintained at 10 °C in the autosampler throughout the analytical process. To minimize signal fluctuations, sample sequences were analyzed continuously in randomized order. The UPLC system was coupled with a Q‐Exactive Plus mass spectrum and electrospray ion source (ESI). Data acquisition was performed in both positive and negative ionization modes. The ESI source parameters were optimized as follows: heater temperature: 300 °C, sheath gas flow rate: 45 arb, Aux gas flow rate: 15 arb, sweep gas flow rate: 1 arb, spray voltage: 3.0 kV, capillary temperature: 350 °C, S‐Lens RF level 50%, with MS1 scan ranges set at 200–1800 *m*/*z*. And lipid identification was performed using “Lipid Search,” a search engine for the identification of lipid species based on MS/MS math. Lipid Search contains more than 30 lipid classes and more than 1 500 000 fragment ions in the database. Both mass tolerance for precursor and fragment were set to 5 ppm.

### Analysis of Mitochondrial Membrane Potential

For assessment of mitochondrial membrane potential (Δ*Ψ*_m_), cells were seeded in 6‐well plates 1 day before the assay to reach ≈70–80% confluence at the time of measurement. On the following day, cells were incubated with 0.5 µm tetramethylrhodamine ethyl ester (MedChemExpress; HY‐D0985A) at 37 °C for 20 min. After treatment, cells were collected, centrifuged at 300 × *g* for 5 min at 4 °C, washed with PBS, and resuspended in 100 µL of PBS. Δ*Ψ*_m_ was then analyzed by flow cytometry (Agilent NovoCyte Advanteon, USA), and data were analyzed using the FlowJo V10 software (Beckton Dickenson).

### Lactate Assay

Lactate levels were measured using the Abbkine CheKine Micro Lactate Assay Kit (Cat. #KTB1100). Standard, blank, and test wells were prepared with 40 µL sample dilution and 10 µL test sample (5× dilution). After adding 50 µL enzyme reagent (except blanks), plates were incubated at 37 °C for 60 min, washed five times with 30× diluted solution, then treated with 50 µL Color Developer A and B and incubated at 37 °C in the dark for 15 min. Finally, 50 µL stop solution was added, and absorbance was read at 450 nm with blanks as calibration.

### Co‐Immunoprecipitation

Co‐IP was conducted using the Pierce Classic Magnetic IP/Co‐IP Kit (Thermo Fisher, USA) according to the manufacturer's instructions. Proteins were extracted using lysis buffer (1861603, Lot# ZJ402422, Thermo Fisher) supplemented with a protease inhibitor cocktail (CWbio, China). Protein lysates were incubated with corresponding antibodies at 4 °C overnight to form immune complexes. Magnetic beads were then added and incubated for 1 h at room temperature, followed by three washes with lysis buffer. Precipitated proteins were eluted with 5× SDS loading buffer and subsequently analyzed by western blotting. To further investigate the interaction between ARL5B and ROCK1, six ROCK1 truncation mutants (ROCK1‐1‐1080, ROCK1‐1‐727, ROCK1‐1‐540, ROCK1‐1‐420, ROCK1‐1096‐1394, and ROCK1‐375‐727) were designed and constructed by Bosheng Biotech (Jinan, China). Wild‐type and truncated ROCK1 plasmids carrying a FLAG tag, together with an HA‐tagged ARL5B plasmid, were transfected into 293T cells, and co‐immunoprecipitation assays were subsequently performed as described above.

### LC‐MS/MS Analysis

For LC‐MS/MS identification of ARL5B interactors, proteins obtained from immunoprecipitation were separated by SDS/PAGE. Gel bands (≈5 × 10 mm) containing target proteins were excised, and the embedded proteins were processed for LC‐MS/MS analysis. Detection was carried out using a Q‐Exactive HF‐X mass spectrometer coupled with an Easy‐nLC 1200 system (Thermo Scientific). LC‐MS analysis was performed by SpecAllyLife Technology Co., Ltd. (Wuhan, China). MS raw data were processed using MaxQuant (V1.6.6) with the Andromeda database search algorithm. Proteins exhibiting a fold change of >4 between bait IP and control samples were identified as potential interactors of the bait protein.

### Protein–Protein Docking

Protein–protein complex structures were predicted using AlphaFold3 (https://alphafoldserver.com/).^[^ ^28^ ^]^ Specifically, the following steps were undertaken: the amino acid sequences of the target proteins were obtained from UniProt (https://www.uniprot.org/) and used as input. The UniProt IDs were Q96KC2 for human ARL5B and Q13464 for human ROCK1, with the detailed protein sequences provided in File S1 (Supporting Information). Multiple sequence alignments (MSAs) were automatically generated by the AF3 pipeline, and predictions were sampled from multiple random seeds, each producing candidate structures through iterative diffusion and denoizing. Following the recommended inference protocol, five independent seeds with five diffusion samples per seed were generated, yielding five structural models. The predicted models were ranked according to the predicted template modeling (pTM) and interface pTM (ipTM) scores, and the highest‐ranked structure was selected for further structural analysis. The final selected model had a pTM score of 0.64, indicating the reliability of the docking result. Visualization of the final models was performed using PyMOL (The PyMOL Molecular Graphics System, Version 3.1.6.1, Schrödinger, LLC).

### In Vivo Experiments

sh‐control‐KYSE150 and sh‐ARL5B‐KYSE150 cells were generated via lentiviral transduction. All animal experiments were approvedby the Institutional Animal Care and Use Committee of Qilu Hospital of Shandong University (approval number: KYLL‐2022(ZM)‐1353). Female BALB/c nude mice aged 6‐8 weeks (Vital River Laboratory Animal Technology Co., Ltd., Beijing, China) were bred under specific pathogen‐free conditions at 24 °C on a 12 h day–night cycle for the establishment of a subcutaneous tumor growth model. Animals housed under similar conditions were randomly divided into the control and experimental groups. Single‐cell suspensions of 1 × 10^6^ cells (sh‐control‐KYSE150 and sh‐ARL5B‐KYSE150) were injected subcutaneously into the left inguinal region of each group of mice. The tumor volume (*V*) was measured every 3 days and was calculated using the following formula: *V* = (*a* × *b* ^2^)/2 (*a* is the long diameter and *b* is the short diameter). Measurements were performed over approximately 3 weeks. To elucidate the functional contribution of ROCK1 in ARL5B‐mediated progression of ESCC, a conditionally regulated ARL5B xenograft model was established. Female BALB/c nude mice (6–8 weeks old) were randomly assigned to four groups (*n* = 5 per group) and subcutaneously injected in the left inguinal region with KYSE150 cell suspensions (1 × 10^6^ cells/100 µL PBS) stably transfected with either pcDNA3.1 (groups 1–2) or pcDNA3.1_ARL5B (groups 3–4). Tumor volume was measured and calculated every 3 days. On day 6 post inoculation, treatment protocols were initiated: groups 1 and 3 received intraperitoneal injections of PBS three times weekly, while groups 2 and 4 were administered fatostatin hydrobromide (25 mg kg^−1^; SelleckChem, Cat. No. S8284) via the same route and frequency, for4 weeks. Then, the mice were sacrificed. Tumors were removed and collected for immunostaining analyses, qRT‐PCR, and western blot analysis.

### Quantification of Neutral Lipids and Triglycerides

Cells were incubated with Nile Red (Beyotime, C2051S‐1, China) for 15 min, fixed with 2% paraformaldehyde, mounted, and counterstained with anti‐fade 4′,6‐diamidino‐2‐phenylindole (DAPI) (Invitrogen). Slides were imaged by a fluorescence microscope system (Echo Revolve, San Diego, CA, USA) and quantified by ImageJ software. The above cells stained were digested with Nile Red and quantified the fluorescence intensity of the lipid droplets by flow cytometry for further quantitative analysis. Triglyceride contents were determined using triglyceride kits (Beyotime, S0219S, China) according to the manufacturers’ protocols.

### Immunofluorescence

Cells were fixed with 4% paraformaldehyde for 15 min at room temperature and then were permeabilized with 0.5% Triton‐X100 for 20 min. After blocking with 5% bovine serum albumin (BSA) for an hour, the cells were incubated at 4 °C overnight with primary antibody (anti‐ARL5B (proteintech, 11694‐1‐AP, 1:1000, China) and anti‐ROCK1 (proteintech, 66782‐1‐Ig, 1:1000, China)). Fluorescein isothiocyanate‐conjugated secondary antibody (Huabio, # HA1122, # HA1121, 1:500, China) was used to incubate with the cells for 60 min at room temperature. After rinses with PBS, the cells were counterstained with DAPI (Solarbio, C0065, Beijing, China) for 15 min in the dark. Images of five randomly selected fields were captured with a fluorescence microscope system (Echo Revolve, San Diego, CA, USA), and fluorescence density was calculated with Image J software (Media Cybernetics Inc., USA).

### Statistical Analysis

All statistical analysis was conducted by R 4.1.3 and GraphPad Prism 9.5 software. Acquired data were certified as normal distribution through Shapiro–Wilk Normality test and homogeneity of variances through the Bartlett test. All the results were presented as the means ± standard deviation (SD)s. Then t‐tests and one‐way analysis of variance (ANOVA) were used for comparisons between two independent samples and comparisons among multiple samples, respectively. The Wilcoxon test was used for nonparametric data. A *p‐*value of <0.05 was considered statistically significant (*p < 0.05; **p < 0.01; and ***p < 0.001). Two‐way ANOVA was used for the experiments with two factors. Pearson correlation was used to calculate the correlation between two or more groups. Kaplan–Meier curve and log‐rank test were used to evaluate survival between different groups.

## Conflict of Interest

The authors declare no conflict of interest.

## Author Contributions

X.Y.M. and Y.F.S. contributed equally to this work. This work was conducted through collaborative efforts from all authors. Y.F.C., R.Q.W., and B.C. served as corresponding authors conceptualizing the study and designing the overall experimental framework while providing comprehensive supervision and final manuscript review. X.Y.M. and Y.F.S. were primarily responsible for conducting the majority of the experimental operations, drafting the initial manuscript and preparing figures. Specimen collection and patient data acquisition were managed by C.H.H. and T.Z.W. Data curation, supplemental experimental work, and results validation were undertaken by H.Y.M. and Z.R.L. Bioinformatics analyses including figure generation were executed by X.Y.Z., D.Z.J., Z.Y.Y., and Z.H.Z. All authors reviewed the manuscript. All authors agree to submit the article for publication.

## Supporting information



Supporting Information

Supplemental Figure 1

Supplemental Figure 2

Supplemental Figure 3

Supplemental Figure 4

Supplemental Table 1

Supplemental Table 2

Supplemental Table 3

Supplemental Table 4

Supporting Information

## Data Availability

The data that support the findings of this study are available from the corresponding author upon reasonable request.
