# Supplementary material for: ARL5B Drives Esophageal Squamous Cell Carcinoma Progression via ROCK1–SREBP1‐Mediated Lipid Metabolic Reprogramming
Source: Adv Sci (Weinh). 2025 Oct 27;13(1):e12895. doi: 10.1002/advs.202512895 (PMC12767069; doi:10.1002/advs.202512895)
Supplement: Supplementary file 1 — Supporting Information [file ADVS-13-e12895-s005.docx]

|Q13464|ROCK1_HUMAN Rho-associated protein kinase 1 OS=Homo sapiens OX=9606 GN=ROCK1 PE=1 SV=1

MSTGDSFETRFEKMDNLLRDPKSEVNSDCLLDGLDALVYDLDFPALRKNKNIDNFLSRYK

DTINKIRDLRMKAEDYEVVKVIGRGAFGEVQLVRHKSTRKVYAMKLLSKFEMIKRSDSAF

FWEERDIMAFANSPWVVQLFYAFQDDRYLYMVMEYMPGGDLVNLMSNYDVPEKWARFYTA

EVVLALDAIHSMGFIHRDVKPDNMLLDKSGHLKLADFGTCMKMNKEGMVRCDTAVGTPDY

ISPEVLKSQGGDGYYGRECDWWSVGVFLYEMLVGDTPFYADSLVGTYSKIMNHKNSLTFP

DDNDISKEAKNLICAFLTDREVRLGRNGVEEIKRHLFFKNDQWAWETLRDTVAPVVPDLS

SDIDTSNFDDLEEDKGEEETFPIPKAFVGNQLPFVGFTYYSNRRYLSSANPNDNRTSSNA

DKSLQESLQKTIYKLEEQLHNEMQLKDEMEQKCRTSNIKLDKIMKELDEEGNQRRNLEST

VSQIEKEKMLLQHRINEYQRKAEQENEKRRNVENEVSTLKDQLEDLKKVSQNSQLANEKL

SQLQKQLEEANDLLRTESDTAVRLRKSHTEMSKSISQLESLNRELQERNRILENSKSQTD

KDYYQLQAILEAERRDRGHDSEMIGDLQARITSLQEEVKHLKHNLEKVEGERKEAQDMLN

HSEKEKNNLEIDLNYKLKSLQQRLEQEVNEHKVTKARLTDKHQSIEEAKSVAMCEMEKKL

KEEREAREKAENRVVQIEKQCSMLDVDLKQSQQKLEHLTGNKERMEDEVKNLTLQLEQES

NKRLLLQNELKTQAFEADNLKGLEKQMKQEINTLLEAKRLLEFELAQLTKQYRGNEGQMR

ELQDQLEAEQYFSTLYKTQVKELKEEIEEKNRENLKKIQELQNEKETLATQLDLAETKAE

SEQLARGLLEEQYFELTQESKKAASRNRQEITDKDHTVSRLEEANSMLTKDIEILRRENE

ELTEKMKKAEEEYKLEKEEEISNLKAAFEKNINTERTLKTQAVNKLAEIMNRKDFKIDRK

KANTQDLRKKEKENRKLQLELNQEREKFNQMVVKHQKELNDMQAQLVEECAHRNELQMQL

ASKESDIEQLRAKLLDLSDSTSVASFPSADETDGNLPESRIEGWLSVPNRGNIKRYGWKK

QYVVVSSKKILFYNDEQDKEQSNPSMVLDIDKLFHVRPVTQGDVYRAETEEIPKIFQILY

ANEGECRKDVEMEPVQQAEKTNFQNHKGHEFIPTLYHFPANCDACAKPLWHVFKPPPALE

CRRCHVKCHRDHLDKKEDLICPCKVSYDVTSARDMLLLACSQDEQKKWVTHLVKKIPKNP

PSGFVRASPRTLSTRSTANQSFRKVVKNTSGKTS

>sp|Q96KC2|ARL5B_HUMAN ADP-ribosylation factor-like protein 5B OS=Homo sapiens OX=9606 GN=ARL5B PE=1 SV=1

MGLIFAKLWSLFCNQEHKVIIVGLDNAGKTTILYQFLMNEVVHTSPTIGSNVEEIVVKNT

HFLMWDIGGQESLRSSWNTYYSNTEFIILVVDSIDRERLAITKEELYRMLAHEDLRKAAV

LIFANKQDMKGCMTAAEISKYLTLSSIKDHPWHIQSCCALTGEGLCQGLEWMTSRIGVR
