# Supplementary figures and images for: ARL5B Drives Esophageal Squamous Cell Carcinoma Progression via ROCK1–SREBP1‐Mediated Lipid Metabolic Reprogramming
Source: Adv Sci (Weinh). 2025 Oct 27;13(1):e12895. doi: 10.1002/advs.202512895 (PMC12767069; doi:10.1002/advs.202512895)

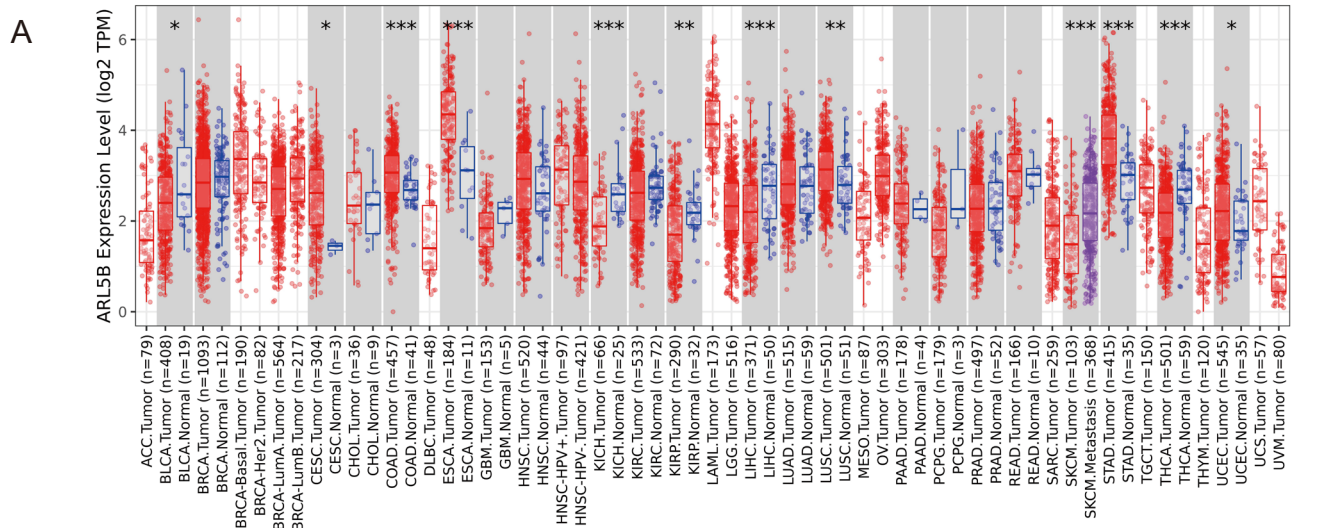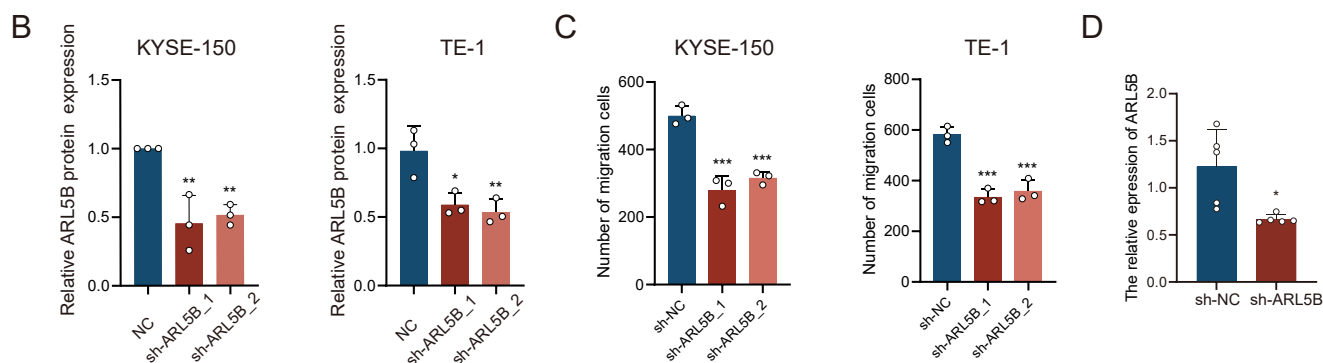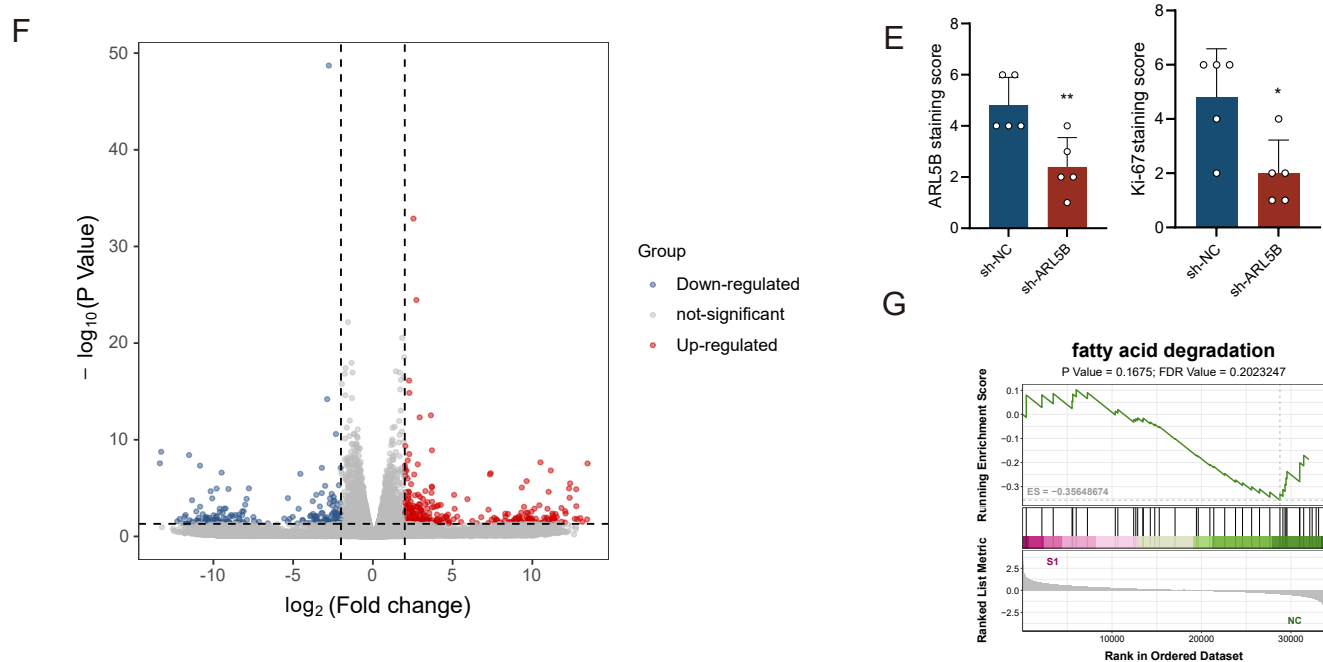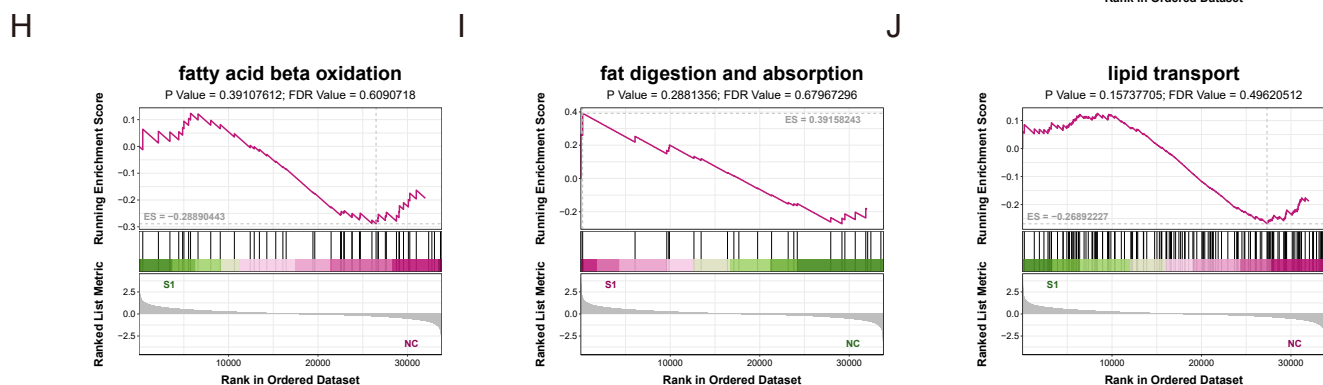

Supplement: Supplementary file 2 — Supplemental Figure 1 [file ADVS-13-e12895-s007.pdf]

A

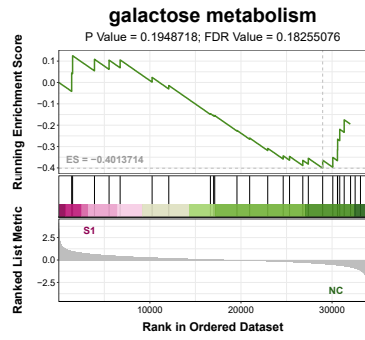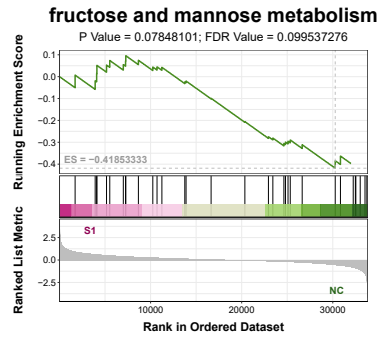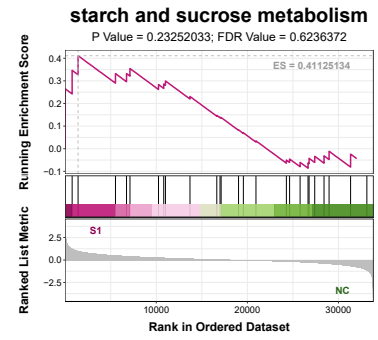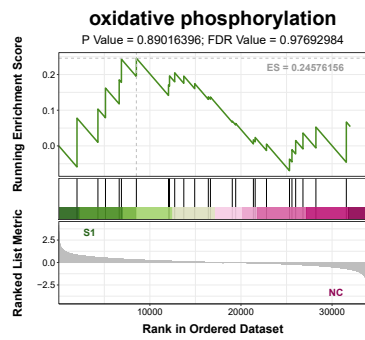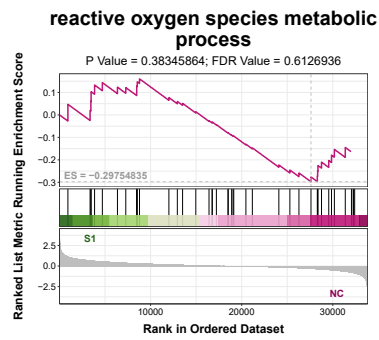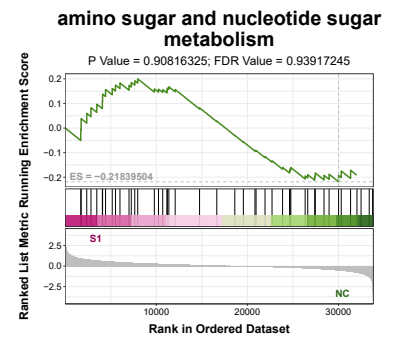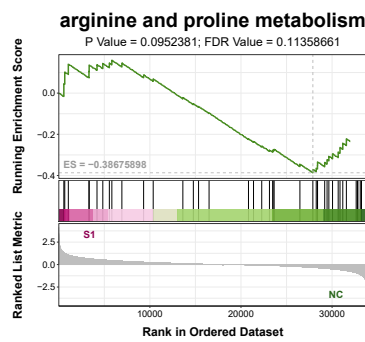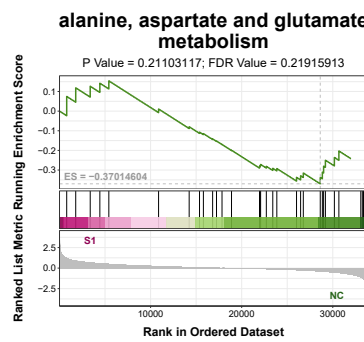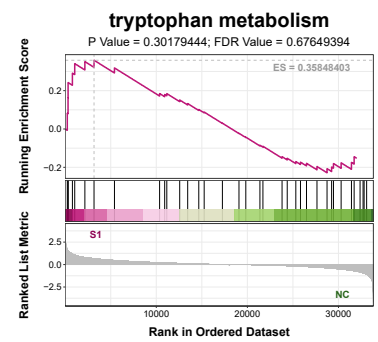

B

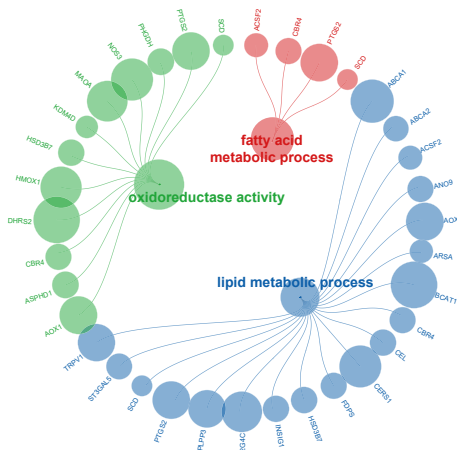

C

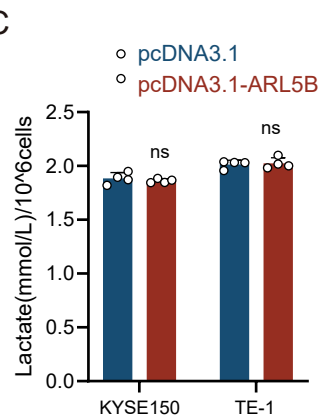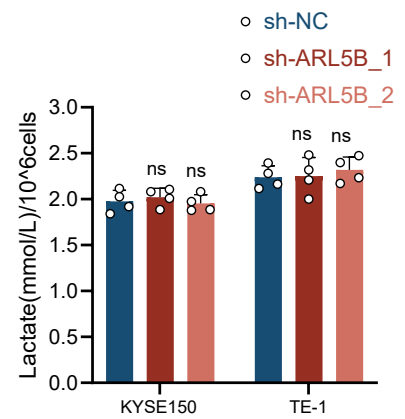

D

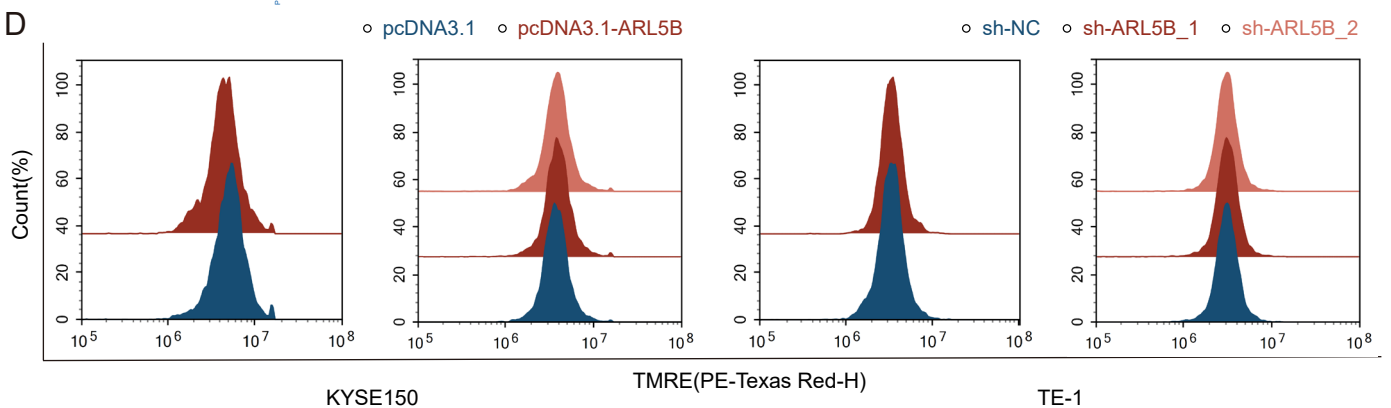

Supplement: Supplementary file 3 — Supplemental Figure 2 [file ADVS-13-e12895-s002.pdf]

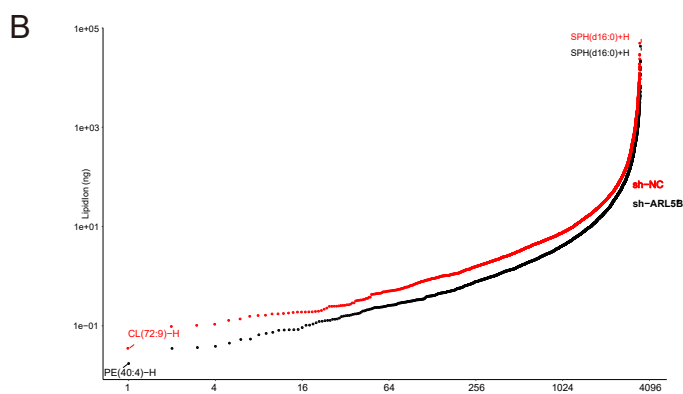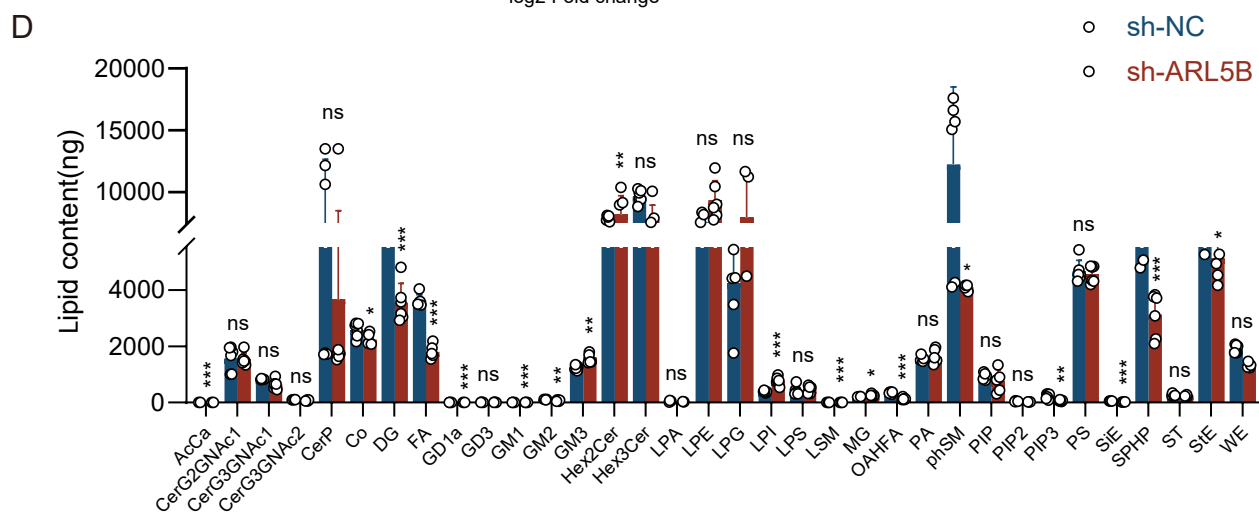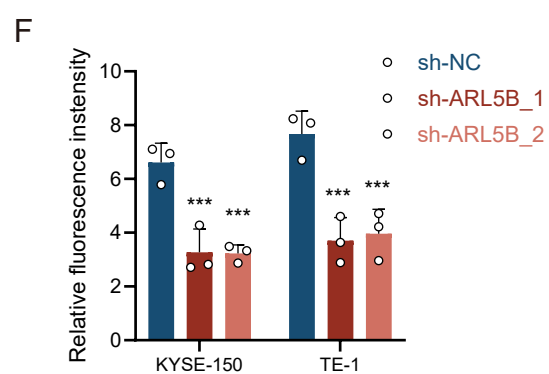

Supplement: Supplementary file 4 — Supplemental Figure 3 [file ADVS-13-e12895-s009.pdf]

A

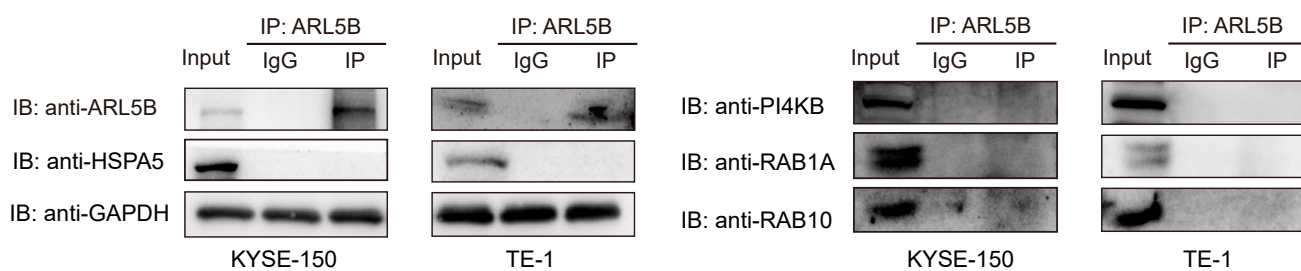

B

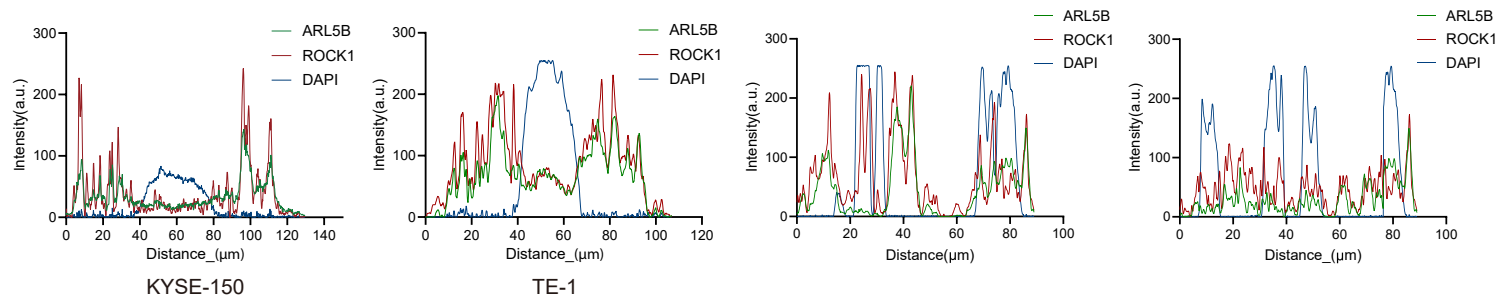

C

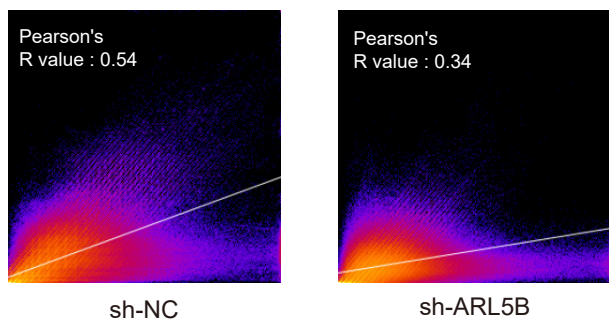

D

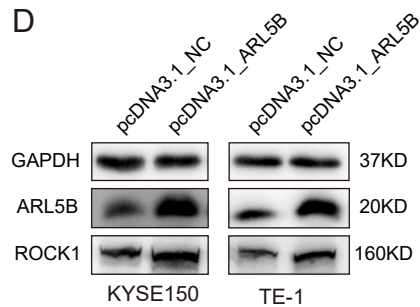

E

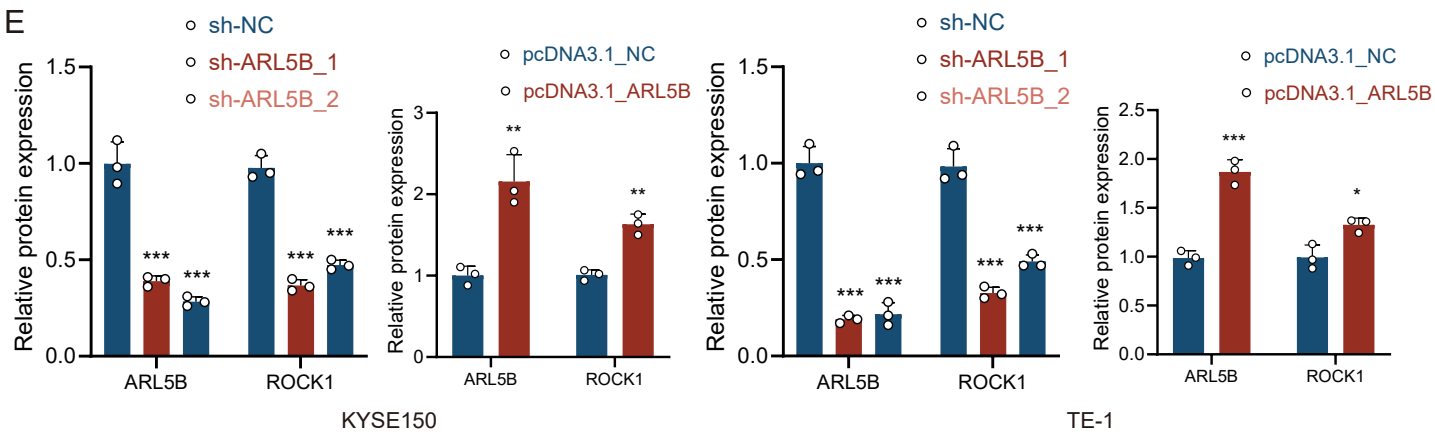

F

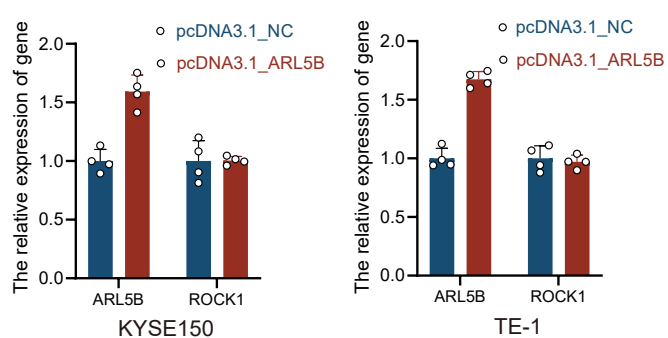

G

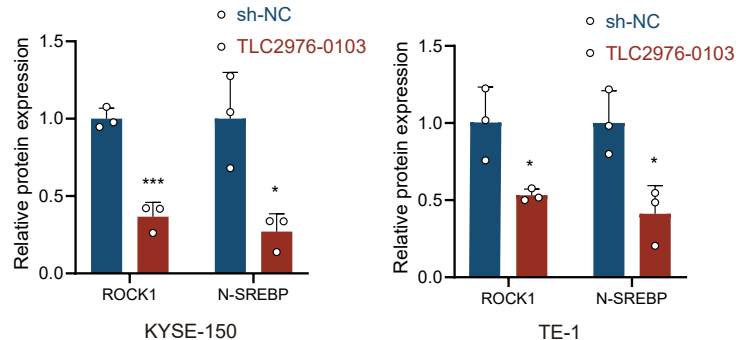

Supplement: Supplementary file 5 — Supplemental Figure 4 [file ADVS-13-e12895-s008.pdf]
