# Supplementary material for: ARL5B Drives Esophageal Squamous Cell Carcinoma Progression via ROCK1–SREBP1‐Mediated Lipid Metabolic Reprogramming
Source: Adv Sci (Weinh). 2025 Oct 27;13(1):e12895. doi: 10.1002/advs.202512895 (PMC12767069; doi:10.1002/advs.202512895)
Supplement: Supplementary file 6 — Supplemental Table 1 [file ADVS-13-e12895-s006.docx]

Supplementary TableS1-patients information

For Immunohistochemistry

| Patient | Gender | Age | T stage | N stage | M stage | TNM stage |
| --- | --- | --- | --- | --- | --- | --- |
| patient1 | female | 53 | T3 | N0 | M0 | II |
| patient2 | male | 68 | T1b | N0 | M0 | I |
| patient3 | male | 46 | T2 | N0 | M0 | II |
| patient4 | male | 68 | T3 | N0 | M0 | II |
| patient5 | male | 72 | T2 | N0 | M0 | II |
| patient6 | female | 52 | T1 | N0 | M0 | I |
| patient7 | female | 64 | T1 | N0 | M0 | I |
| patient8 | male | 74 | T1 | N0 | M0 | I |
| patient9 | female | 62 | T1 | N0 | M0 | I |
| patient10 | male | 64 | T1 | N0 | M0 | I |
| patient11 | female | 68 | T2 | N0 | M0 | II |
| patient12 | female | 42 | T2 | N0 | M0 | II |
| patient13 | female | 66 | T2 | N0 | M0 | II |
| patient14 | female | 66 | T2 | N0 | M0 | II |
| patient15 | female | 59 | T2 | N0 | M0 | II |
| patient16 | male | 76 | T4 | N0 | M0 | III |
| patient17 | male | 54 | T4 | N1 | M0 | III |
| patient18 | male | 55 | T4 | N1 | M0 | III |
| patient19 | male | 45 | T4 | N1 | M0 | III |
| patient20 | male | 58 | T4 | N0 | M0 | III |
| patient21 | male | 67 | T4 | N0 | M0 | IVA |
| patient22 | male | 53 | T4 | N3 | M0 | IVA |
| patient23 | male | 52 | T4 | N3 | M0 | IVB |
| patient24 | male | 61 | T4 | N3 | M0 | IVB |
| patient25 | male | 57 | T4 | N3 | M0 | IVB |

For western blot

| Patient | Gender | Age | T stage | N stage | M stage | TNM stage |
| --- | --- | --- | --- | --- | --- | --- |
| patient1 | male | 78 | T3 | N0 | M0 | II |
| patient2 | male | 70 | T3 | N0 | M0 | II |
| patient3 | male | 68 | T1b | N0 | M0 | I |
| patient4 | female | 72 | T4a | N1 | M0 | IVA |
| patient5 | male | 64 | T3 | N0 | M0 | II |
| patient6 | male | 73 | T3 | N1 | M0 | III |
| patient7 | male | 61 | T3 | N1 | M0 | III |
| patient8 | female | 81 | T3 | N1 | M0 | III |
| patient9 | male | 61 | T3 | N0 | M0 | II |
| patient10 | male | 67 | T2 | N0 | M0 | II |
| patient11 | male | 68 | T1b | N0 | M0 | I |
| patient12 | male | 65 | T2 | N0 | M0 | II |
