# Supplementary material for: ARL5B Drives Esophageal Squamous Cell Carcinoma Progression via ROCK1–SREBP1‐Mediated Lipid Metabolic Reprogramming
Source: Adv Sci (Weinh). 2025 Oct 27;13(1):e12895. doi: 10.1002/advs.202512895 (PMC12767069; doi:10.1002/advs.202512895)
Supplement: Supplementary file 7 — Supplemental Table 2 [file ADVS-13-e12895-s003.docx]

Supplementary TableS2: The sequence of primers

| Primer | Species | Forward/ Reverse | Sequence (5'-3') |
| --- | --- | --- | --- |
| GAPDH | homos | Forward | GCACCGTCAAGGCTGAGAAC |
|  |  | Reverse | TGGTGAAGACGCCAGTGGA |
| β-tubulin | homos | Forward | CATGGACTCTGTTCGCTCAGG |
|  |  | Reverse | CCTTTGGCCCAGTTGTTACCT |
| Vinculin | homos | Forward | TCGTCCGGGTTGGAAAAGAG |
|  |  | Reverse | GACCTCAGCCTCATCGAAGG |
| ARL5B | homos | Forward | AGTGGGACTGGATAATGCAGGG |
|  |  | Reverse | ATCGCAGAGACTCCTGACCACC |
| SCD1 | homos | Forward | TCTAGCTCCTATACCACCACCA |
|  |  | Reverse | TCGTCTCCAACTTATCTCCTCC |
| ACLY | homos | Forward | AGGGAGCTTGGTTTGATCCG |
|  |  | Reverse | GCCAATGCCCATCTCTTCCT |
| FASN | homos | Forward | GGTCTACTACGCCTCCCTCA |
|  |  | Reverse | GTTGGAAGGCACATCCCAGA |
| ACACA | homos | Forward | AGGAGCTGTCTATTCGGGGT |
|  |  | Reverse | GGTCGCTCAGCCTGTACTTT |
| HMGCR | homos | Forward | GATAGGAACGGTGGGTGGTG |
|  |  | Reverse | CCACACACAATTCGGGCAAG |
| LDL | homos | Forward | GGGCGACAGATGCGAAAGAA |
|  |  | Reverse | CCATCGCAGACCCACTTGTA |
| CD36 | homos | Forward | TGCAAAGAAGGGAGACCTGTG |
|  |  | Reverse | GTTGACCTGCAGCCGTTTTG |
| SREBF1 | homos | Forward | TTCTTCGTGGATGGGGACTG |
|  |  | Reverse | GTCACACAGTTCAGTGCTCG |
| ROCK1 | homos | Forward | TTGCTGTGCTGTGAATGA |
|  |  | Reverse | GGTAATGGTGATGAGTCTGA |
