# Supplementary material for: ARL5B Drives Esophageal Squamous Cell Carcinoma Progression via ROCK1–SREBP1‐Mediated Lipid Metabolic Reprogramming
Source: Adv Sci (Weinh). 2025 Oct 27;13(1):e12895. doi: 10.1002/advs.202512895 (PMC12767069; doi:10.1002/advs.202512895)
Supplement: Supplementary file 8 — Supplemental Table 3 [file ADVS-13-e12895-s004.docx]

Supplementary TableS3: the information of primary antibodies

| **Antibody** | **Host Species** | **Company** | **Catalog Number** | **Dilution ration** | **Application** |
| --- | --- | --- | --- | --- | --- |
| Vinculin | Rabbit | Proteintech | 66305-1-Ig | 1: 5000 | Western Blot |
| GAPDH | Rabbit | Cell signaling | #2118 | 1: 5000 | Western Blot |
| ARL5B | Rabbit | Cusabio technology Antibody | #CSB-PA002091GA01HU | 1:100 | IHC |
| ARL5B | Rabbit | Proteintech | 11694-1-AP | 1: 1000 | Western Blot, IF |
| ARL5B | Mouse | Santa Cruz Biotechnology | sc-393511 | - | CoIP |
| ARL5B | Rabbit | Affinity | DF12834 | 1:500 | Immunofluorescence |
| SREBP1 | Rabbit | HuaBio | HA500210 | 1: 1000 | Western Blot |
| SREBF1 | Rabbit | Cell signaling | #95879 | 1: 1000 | Western Blot |
| SREBF1 | Rabbit | Proteintech | 14088-1-AP | 1: 500 | Immunofluorescence |
| ROCK1 | Rabbit | Proteintech | 21850-1-AP | 1: 1000 | Western Blot |
| ROCK1 | Mouse | Proteintech | 66782-1-Ig | 1:500 | Immunofluorescence |
| Calnexin | Rabbit | Proteintech | 66903-1-Ig | 1:500 | Immunofluorescence |
| GM130 | Rabbit | Abclonal | A5344 | 1:500 | Immunofluorescence |
| GM130 | Mouse | Proteintech | 66662-1-Ig | 1:500 | Immunofluorescence |
| PIK4B | Rabbit | Proteintech | 13247-1-AP | 1: 1000 | Western Blot |
| BiP | Rabbit | Cell signaling | #3177T | 1: 1000 | Western Blot |
| RAB10 | Rabbit | Cell signaling | #8127T | 1: 1000 | Western Blot |
| RAB1A | Rabbit | Proteintech | 11671-1-AP | 1: 1000 | Western Blot |
