# Supplementary material for: ARL5B Drives Esophageal Squamous Cell Carcinoma Progression via ROCK1–SREBP1‐Mediated Lipid Metabolic Reprogramming
Source: Adv Sci (Weinh). 2025 Oct 27;13(1):e12895. doi: 10.1002/advs.202512895 (PMC12767069; doi:10.1002/advs.202512895)
Supplement: Supplementary file 10 — Supporting Information [file ADVS-13-e12895-s010.docx]

**Supplementary Figure S1**

A. Bioinformatic analysis of ARL5B expression across malignancies in the TCGA database, showing associations with various malignant tumors. B. Western blot analyses with grayscale quantification confirmed efficient suppression of ARL5B protein expression. C. Quantitative statistical data from Transwell invasion assays in KYSE150 and TE-1 cells with or without ARL5B knockdown. D. Quantification of immunoblot showing ARL5B protein in xenograft tumor tissues from NC and ARL5B-knockdown groups. E. Immunohistochemical (IHC) staining of ARL5B and Ki-67 in xenograft tumor tissues, quantified using H-scores. F. Volcano plot of RNA-sequencing data from KYSE150 cells following ARL5B knockdown. G-J. Gene Set Enrichment Analysis (GSEA) enrichment plots for KEGG pathways: Fatty acid degradation (F), fatty acid beta oxidation (G), fat digestion and absorption (H) and lipid transport (I). Data are presented as mean SD. *, *p* < 0.05; **, *p* < 0.01; ***, *p* < 0.001; ns, no significance.

**Supplementary Figure S2**

A. GSEA plots of glucose metabolism, oxidative phosphorylation, reactive oxygen species metabolism, and amino acid metabolic pathways in ARL5B-knockdown cells. B. Bubble plot of differentially expressed genes associated with lipid metabolic processes. C. Measurement of cellular lactate levels in KYSE150 and TE-1 cells with ARL5B overexpression or knockdown. D. Flow cytometric analysis of mitochondrial membrane potential (ΔΨm) using TMRE staining in KYSE150 and TE-1 cells with ARL5B overexpression or knockdown. Data are presented as mean SD. *, *p* < 0.05; **, *p* < 0.01; ***, *p* < 0.001; ns, no significance.

**Supplementary Figure S3**

A. Principal component analysis showing clustering patterns between control and ARL5B-knockdown groups. B. Dynamic distribution range of lipid content, each dot in the figure represents a lipid molecule. C. Volcano plot of LC-MS/MS lipidomics data from KYSE150 cells with or without ARL5B knockdown. Red: upregulated lipid ions; purple: downregulated lipid ions; black: unchanged lipid ions. D. Stacked bar graph shows quantified lipid classes based on analysis of LC-MS/MS lipidomic profiling data (n=6). E. Lipid content of top 10 significant TG species in the control and ARL5B knockdown group. F. Quantification of fluorescence intensity from Nile Red-stained neutral lipids in control versus ARL5B-knockdown KYSE150 and TE-1 cells. Data are presented as mean SD. *, *p* < 0.05; **, *p* < 0.01; ***, *p* < 0.001; ns, no significance.

**Supplementary Figure S4**

A. Co-IP assays between ARL5B and candidate proteins (PI4KB, HSPA5, RAB10, RAB1A). B. Immunofluorescence co-localization of ARL5B (green) and ROCK1 (red) in KYSE150 and TE-1 cells (upper) and xenograft tumor tissues (lower). C. Pearson’s correlation coefficients quantifying ARL5B and ROCK1 co-localization in tumor tissues. D. Immunoblot analysis of ROCK1 protein expression in ARL5B-overexpressing cells. E. mRNA expression levels of ROCK1 in ARL5B-overexpressing cells. F. Quantification of ARL5B and ROCK1 protein levels by immunoblot after ARL5B knockdown or overexpression, corresponding to Figure 5A and Supplementary Figure S4D. G. Quantification of immunoblot showing N-SREBP1 expression in KYS
